# The effects of combined aerobic and resistance exercise on metabolic blood biomarkers and quality of life in patients with breast cancer: a systematic review meta-analysis

**DOI:** 10.3389/fonc.2026.1900945

**Published:** 2026-09-11

**Authors:** Muhammad Ibrar Ahmad, Ali Raza, Jie Bao, Xiaolin Chen, Yaqin Li, Yang Li, Yunhang Lu

**Affiliations:** 1 School of Physical Education and Sports Science, Soochow University, Suzhou, China; 2 Department of Sports Sciences and Physical Education, University of the Punjab, Lahore, Punjab, Pakistan; 3 First Teaching Hospital of Tianjin University of Traditional Chinese Medicine, Tianjin, China; 4 National Clinical Research Center for Chinese Medicine, Tianjin, China; 5 The Fourth People’s Hospital of Chengdu, Chengdu, China; 6 School of Physical Education, Anshan Normal University, Anshan, Liaoning, China

**Keywords:** aerobic exercise, breast cancer, combined exercise, metabolic biomarkers, quality of life

## Abstract

**Objective:**

The systematic review and meta-analysis aimed to evaluate the effects of combined aerobic and resistance exercise (CE) on metabolic blood biomarkers (MBB), quality of life (QoL), and QoL subscales in women with breast cancer (BC). A secondary objective was to investigate whether exercise dose characteristics influenced these outcomes.

**Methods:**

Randomized controlled trials (RCTs) were comprehensively searched for studies investigating the effects of CE on BC patients across four databases (PubMed, Cochrane, MEDLINE, and Web of Science). The standardized mean difference (Hedges’ *g*) was calculated using Comprehensive Meta-Analysis (CMA V4), and the risk of bias was assessed using RevMan 5.4.

**Results:**

Of the 2,654 records identified, 37 RCTs met the inclusion criteria, with 24 RCTs focusing on overall QoL, nine evaluating FACT-B, and six to nine RCTs assessing MBB outcomes. CE exercise significantly improved overall QoL (Hedges’ *g* = 1.10; 95% CI: 0.72, 1.48; *p* < 0.0001), FACT-B (Hedges’ *g* = 1.14; 95% CI: 0.46, 1.81; *p* = 0.001), HDL (Hedges’ *g* = 0.828; 95% CI: 0.051, 1.606; *p* = 0.037), HOMA-IR (Hedges’ *g* = − 0.581, 95% CI: − 1.059, − 0.103; *p* = 0.017), and DBP (Hedges’ *g* = − 0.39; 95% CI: − 0.786, − 0.0008; *p* = 0.045). No significant effects were observed for glucose, insulin, triglycerides, LDL, or SBP. Secondary analyses further suggested that higher exercise doses may be associated with greater improvements in these outcomes than shorter exercise doses.

**Conclusion:**

CE was associated with significant improvements in MBB, QoL, and QoL subscales in breast cancer patients; however, substantial heterogeneity should be considered when interpreting these findings. Exercise dose characteristics may influence QoL and selected MBB outcomes. Further studies are needed to evaluate the optimal dose–response effects of CE in BC patients.

**Systematic Review Registration:**

https://www.crd.york.ac.uk/PROSPERO/view/CRD420251032722, identifier CRD420251032722.

## 1 Introduction

Breast cancer (BC) represents the most frequently diagnosed cancer among women globally, with Asia reporting the highest incidence (985, 817 cases), followed by Europe (557, 532) and North America (306, 307) (1). In 2022, breast cancer was identified as the second primary cause of cancer-related mortality among women, resulting in 670,000 deaths (2). Alongside the increasing incidence and mortality rates, metabolic syndrome represents a significant risk factor for the occurrence and development of breast cancer.

Metabolic syndrome, first defined in 2001, has emerged as a significant public health issue, particularly in nations characterized by high obesity prevalence and Western dietary patterns (3, 4). It comprises multiple interrelated risk factors, including visceral fat accumulation, elevated blood glucose, dyslipidemia, insulin resistance assessed using the Homeostatic Model Assessment of Insulin Resistance (HOMA-IR), elevated triglycerides (TG), reduced insulin sensitivity, and high blood pressure (BP), which may influence the recurrence and progression of BC (5). Recent evidence indicates that metabolic syndrome contributes to a higher likelihood of BC development, recurrence, and mortality (6, 7). Prior research has also shown that women with metabolic syndrome have a higher risk of developing breast cancer (8–10).

Despite these risks, advancements in diagnostic methods and medical technologies, including chemotherapy and radiotherapy, have substantially enhanced the treatment of breast cancer. However, these treatment modalities, whether used individually or in combination, frequently lead to numerous side effects in BC patients and survivors. These adverse effects include metabolic health issues, physical impairments, fertility issues, anxiety, depression, and disruptions in social and work activities, which collectively lead to a decreased quality of life (QoL) (11, 12). In clinical practice, the administration of pharmacological agents and cardioprotective therapies is frequently utilized to reduce treatment-related toxicities (13). However, some supportive pharmacological treatments may produce additional adverse effects, such as hypotension, fatigue, gastrointestinal disturbances, and potential drug-related toxicities (14). Therefore, addressing these side effects is a critical component of BC care.

Given these limitations, modifications in habits and lifestyle have become essential for preventing adverse metabolic outcomes and improving QoL. In this context, incorporating exercise into daily life has been associated with a reduced risk of BC relapse (15) and is widely recognized in modern medicine as a complementary therapy to alleviate treatment-related side effects. Encouragingly, many investigations have indicated that exercise may enhance tumor-specific immune responses without negatively affecting the immune system in cancer survivors, particularly those with BC (16–18), and could be beneficial for improving metabolic blood markers and QoL. Structured and supervised exercise interventions may help alleviate the psychological and physical difficulties faced by BC patients (19–21).

According to the American College of Sports Medicine (ACSM) recommendations, aerobic exercise can improve health-related QoL, physical function, sleep quality, insulin sensitivity, and cardiorespiratory fitness while also reducing anxiety and depression. In contrast, resistance training has proven beneficial for improving QoL, attenuating lymphedema, and enhancing aspects of bone health. Recent research has explored the integration of combined aerobic and resistance exercises (CE), with recommendations encouraging cancer patients to engage in such programs (17). A meta-analysis has demonstrated that CE is superior to each modality alone in enhancing metabolic health outcomes and QoL in BC patients (21, 22). However, these studies had several limitations that could affect the results, including low study quality, unexplained heterogeneity, limited focus on QoL subdomains, and limited evaluation of exercise dose parameters, such as intervention duration, exercise frequency, and session duration.

Although evidence suggests that exercise is an effective adjunct to conventional treatment across various disease fields, especially for treating certain cancers (23), important gaps remain in current research on women with breast cancer. In particular, there is insufficient information regarding how differences in exercise dose, such as overall exercise duration, frequency, and session duration, influence health-related recovery outcomes. Given that physiological responses may differ based on exercise dose, there is a need for systematic investigation into how exercise promotes the recovery of women with breast cancer. Additionally, an attempt should be made to identify evidence-based guidelines regarding the overall exercise duration and frequency needed to maximize therapeutic benefits, mitigate adverse effects associated with treatment, and prevent metabolic complications.

Therefore, this systematic review and meta-analysis provides new perspectives on the current literature by evaluating the influence of CE on metabolic blood markers, overall QoL, and QoL subscales in BC patients. The QoL subscales assessed included physical functioning (PF), role functioning (RF), social functioning (SF), cognitive functioning (CF), and emotional functioning (EF). The second aim of this research was to evaluate exercise dose parameters, including intervention duration (duration/week), session duration (min/day), and frequency (sessions/week).

## 2 Method

This meta-analysis followed the PRISMA 2020 checklist to ensure methodological rigor and transparency. This review was registered on PROSPERO, with registration number CRD420251032722.

### 2.1 Search strategy

A comprehensive search was conducted across four databases: PubMed, MEDLINE, Web of Science, and the Cochrane Library. Boolean operators (e.g., AND, OR) were used to identify studies using the following keywords: (“breast cancer” OR “breast neoplasm” OR “breast carcinoma” OR “breast tumor” OR “female” OR “survivor”) AND (“physical activity” OR “exercise” OR “aerobic exercise” OR “resistance exercise” OR “combined training” OR “strength training” OR “supervised exercise”) AND (“metabolic syndrome” OR “metabolic markers” OR “triglycerides” OR “cholesterol” OR “blood glucose” OR “insulin resistance” OR “blood pressure” OR “quality of life” OR “mental health” OR “functional status”). The databases were searched from inception until August 2025. The detailed search strategy for each database was provided in **Supplementary Material S1**. A manual search was also conducted by the second author by reviewing the references of published studies to ensure a rigorous search strategy and minimize bias. Duplicate removal and title/abstract screening were performed in EndNote 2025, and then two authors manually screened the studies according to the predetermined inclusion criteria.

### 2.2 Eligibility criteria and study selection

Studies included in this analysis adhered to the PICO framework. The inclusion criteria were defined as follows: population: women diagnosed with breast cancer; intervention: combined aerobic and resistance exercise performed at least 4 weeks; comparison: combined aerobic and resistance exercise vs. usual care; outcomes: studies reporting at least one of the following outcomes were included: metabolic blood biomarkers such as triglycerides, low-density lipoprotein cholesterol (LDL), high-density lipoprotein protein cholesterol (HDL), fasting glucose, insulin, systolic blood pressure (SBP), diastolic blood pressure (DBP), and HOMA-IR; quality of life scales such as SF-36, QLQ-C30, MQoL, FACT-G, and Functional Assessment of Cancer Therapy-Breast (FACT-B) and their subscales PF, RF, SF, EF, and CF. Studies were included if they were RCTs and were published in English.

Exclusion criteria were set as follows: studies involving cancers other than breast cancer, such as prostate cancer, and male breast cancer were excluded; studies including aerobic or resistance exercise alone or other individual exercise modalities, such as strength training or yoga, were excluded; studies with only one group, such as an experimental group only, were excluded; studies reporting only inflammatory marker outcomes, such as CRP, or other quality-of-life scales mot listed above were excluded; and review articles, theses/dissertations, or abstracts were excluded.

### 2.3 Data extraction

Two reviewers independently screened the titles and abstracts to determine whether studies met the inclusion criteria. The full texts were reviewed afterward, and discrepancies were resolved by consulting a third, experienced author. Means, SDs, and sample sizes were collected from the included studies to conduct the analysis. If any data were missing, emails were sent to the corresponding and original authors. According to the Cochrane Handbook (24), data were converted to mean ± SD. If data were presented in another form, such as median ± standard error, then relevant information from the studies was extracted. The extracted information comprised the author’s name, country of origin, year of publication, age, sample sizes (experimental and usual care groups), study design, interventions, exercise duration, session length, frequency, cancer stage, menopausal status, and outcomes.

### 2.4 Potential risk, certainty of evidence, and study quality assessment

The Cochrane Collaboration tool was employed to examine the risk of bias and methodological rigor of the included studies (25). This tool was selected because all included studies used a randomized controlled trial design. This method is recognized as a robust and comprehensive tool for identifying potential biases. This tool evaluates the quality of studies across different domains such as random sequence generation, allocation concealment, blinding of participants and personnel, blinding of outcome assessors, completeness of outcome data, and selective outcome reporting. The risk was assessed by the first author as low, high, or unclear and cross-checked by the second author. Any remaining discrepancies were resolved through consultation with a third experienced author.

The certainty of the evidence for the main outcomes was assessed using the Grading of Recommendations Assessment, Development and Evaluation (GRADE). The certainty of the evidence was rated across five domains: risk of bias, inconsistency, indirectness, imprecision, and publication bias. The certainty of the evidence was rated for each outcome as high, moderate, low, or very low. The GRADE assessment was conducted for the primary outcomes: quality of life, QoL subscales, and metabolic blood biomarkers.

### 2.5 Subgroup analyses

We performed subgroup analyses based on clinically and methodologically relevant characteristics to explore potential sources of heterogeneity across the included studies. These were (1) exercise supervision status, (2) treatment status of participants, and (3) QoL assessment scales. Exercise supervision was classified into supervised exercise interventions and hybrid/home-based exercise interventions. Treatment status was classified into the active-treatment phase and the post-treatment phase according to the information reported in the included studies. QoL outcomes were also evaluated through different measurement instruments including EORTC QLQ-C30, FACT-G, SF-36, and MQoL. These subgroup analyses were performed to assess whether heterogeneity in intervention delivery, clinical status, or outcome measurement contributed to the observed heterogeneity.

Subgroup analyses according to menopausal status and cancer stage were considered. However, these analyses were not performed because the included studies often reported mixed populations or did not provide sufficient category-specific data to produce reliable pooled estimates.

### 2.6 Statistical analysis

In Comprehensive Meta-Analysis software (CMA V4), the standardized mean difference (Hedges’ *g*) was used to conduct all statistical analyses across studies (CE and usual care groups), and Review Manager 5.3 was used to assess the study quality. The decision to use Hedges’ *g* for statistical analysis was driven by differences in measurement scales across studies and by the inclusion of a small number of studies in some analyses. Due to variability in scales across studies, the 95% confidence interval (CI) and Hedges’ *g* were used to estimate the effect size (24). Therefore, the random-effects model was used across all analyses, and the effect size was defined as 0.2 (small), 0.5 (moderate), and 0.8 (large). Heterogeneity was assessed using *I*^2^ and Cochrane’s *Q*. The *I*^2^ was interpreted as low heterogeneity if *I*^2^ < 25%; moderate heterogeneity was considered when *I*^2^ = 25-49%; significant heterogeneity was considered when *I*^2^ = 50%–75%; and high heterogeneity was considered when *I*^2^ > 75% (25). The threshold *p* < 0.10 was set for statistical significance of high heterogeneity across studies.

In addition to the primary meta-analyses, secondary exploratory subgroup analyses were conducted to investigate whether exercise-related characteristics contributed to variability in treatment effects. These analyses were based on the duration of the intervention, frequency of exercise, and duration of session. The cut-offs for > 12 weeks, > 3 sessions/week, and > 60 min/session were selected according to the distribution of exercise characteristics across the included studies. These cut-offs were selected to ensure a sufficient number of studies in each subgroup and to facilitate meaningful exploratory comparisons. Previous cancer exercise guidelines have also emphasized the importance of exercise frequency, duration, and session length as key prescription variables; however, an optimal dose–response relationship remains uncertain (17, 26).

Funnel plots and Egger’s test were used to assess publication bias across studies (27, 28). Moreover, trim-and-fill analysis was used to adjust for missing studies. Sensitivity analysis was performed to assess the reliability of the results and identify the source of heterogeneity across studies by removing each study in turn from the analysis.

## 3 Results

### 3.1 Search results

**Figure 1:**
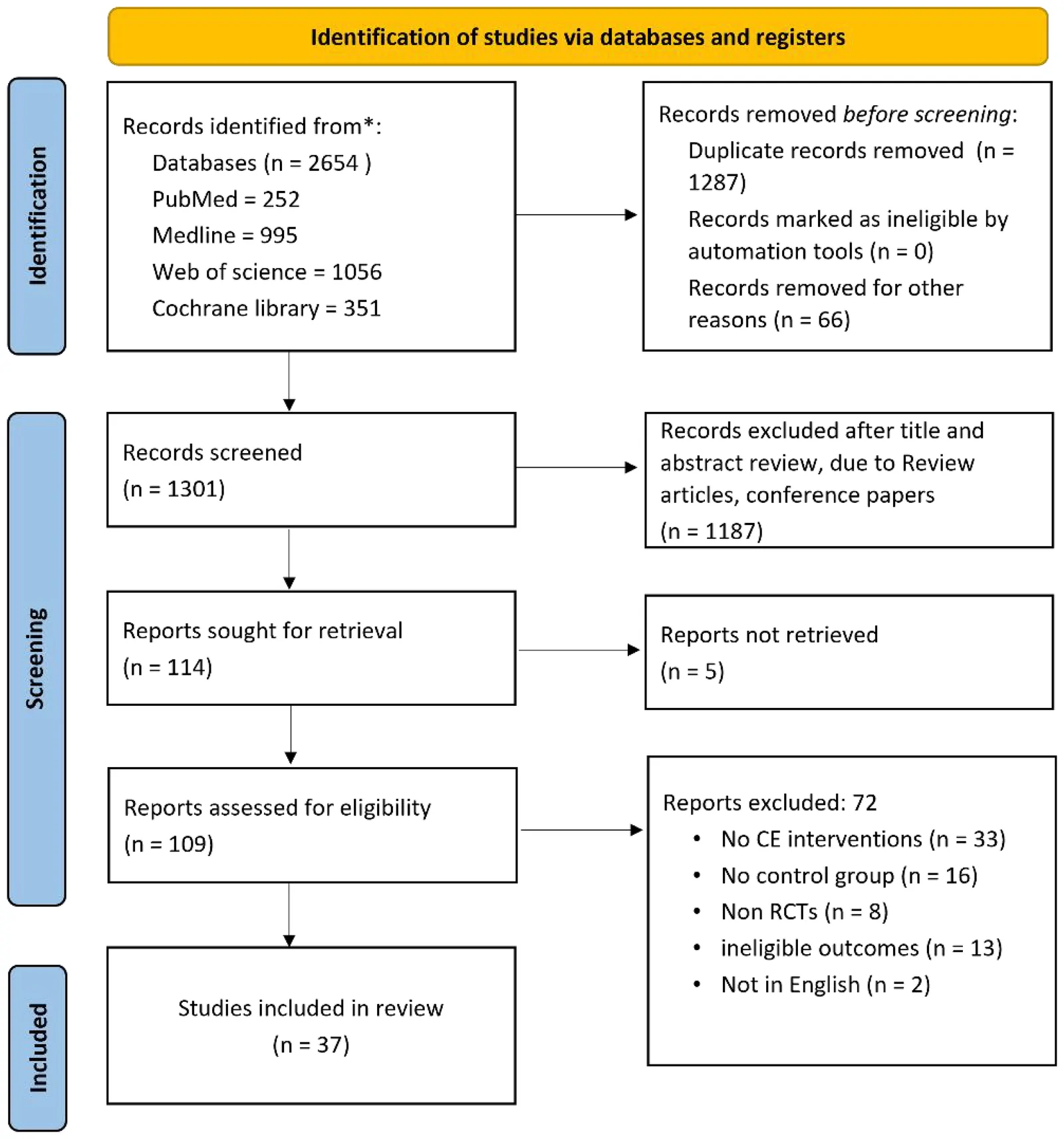
Flow diagram of the literature search and study selection process.

Initially, 2,654 records were identified from four databases: PubMed = 252, MEDLINE = 995, Web of Science = 1,056, and Cochrane Library = 351. Using EndNote, 1,287 articles were removed due to duplicates, and 66 were removed for other reasons. A total of 1,301 articles were considered for screening, and two authors independently screened the titles and abstracts and excluded 1,187 records due to irrelevance or because they were review articles. Five studies could not be retrieved, leaving 109 studies to be assessed for eligibility. After reading the full-text articles, 72 studies were excluded for the reasons described in **Figure 1**. Finally, 37 studies met the criteria for inclusion in the analysis (29–65).

### 3.2 Study characteristics

The 37 included studies are described in **Table 1**. All included studies were RCTs evaluating the effects of combined aerobic and resistance exercise on metabolic outcomes and quality of life in breast cancer patients. Intervention duration ranged from 4 to 52 weeks, exercise frequency ranged from one to four sessions per week, and the session duration of the included studies ranged from 30 to 180 min. The studies were conducted across various regions, including the USA, UK, Spain, Korea, Brazil, Iran, Turkey, the Netherlands, Australia, Portugal, Italy, China, and Poland. The included studies varied in terms of breast cancer stage, menopausal status, and treatment phase. Some studies included patients undergoing chemotherapy and radiotherapy, while others targeted survivors. The outcomes analyzed in this meta-analysis were summarized in **Table 1**.

**Table 1:** Included study characteristics.

Study	Year	Country	Age	n = (CE, UC)	Study Design	D/W	S/W	Min/D	Stage BC	Menopausal status	Treatment	Outcomes Analyzed
Amirabbas et al. (29)	2020	Iran	≥30	38 (20,18)	RCTs	8w	3s	50min	I	Post-m	Survivors, Chemo/RT	QoL (EROTC QLQ-C30), PF, EF
Antunes et al. (30)	2024	Portugal	49.66 ± 9.43 / 51.02 ± 9.54	93 (47,46)	RCTs	20w	3s	60min	NS	Post-m	During Chemo	QoL (EROTC QLQ-C30), PF, RF, SF, CF, EF
Aydin et al. (31)	2021	Turkey	45.0 ± 2.2	48 (24,24)	RCTs	12w	3s	50min	NS	NS	During Chemo	QoL (EROTC QLQ-C30), PF, RF, SF, CF, EF
Baglia et al. (32)	2019	USA	62.0 ± 7.0 /60.5 ± 7.0	121 (61,60)	RCTs	48w	2s	50min	I-III	Post-m	Survivors, Chemo & RT	QoL (FACT-G, FACT-B), PF, SF, EF
Basen et al. (33)	2020	USA	49.6 ± 13.3/ 49.2 ± 9.2	37 (19,18)	RCTs	24w	1s	60min	II-III	Pre-m Post-m,	During Chemo	QoL (SF-36), PF, RF, SF, EF
Brown et al. (34)	2021	USA	59.1 ± 8.1 / 59.0 ± 8.5	177 (87,90)	RCTs	52w	2s	60min	I-III	Post-m	Survivors, Chemo/RT	QoL (SF-36), PF, RF, SF, EF
Campbell et al. (35)	2005	UK	48.0 ± 10/ 47 ± 5	22 (12,10)	RCTs	12w	2s	60min	I-III	NS	During Chemo/RT	QoL (SF-36, FACT-B), PF
Casla et al. (36)	2015	Spain	45.91 ± 8.21 / 51.87 ± 8.21	94 (47,47)	RCTs	12w	2s	60min	I-III	NS	Survivors, Chemo/RT	QoL (SF-36), PF, SF, CF, EF
Chang et al. (37)	2020	Korea	51.4 ± 7.5 / 50.0 ± 6.1	46 (23,23)	RCTs	12w	3s	40min	I-III	NS	Survivors, Chemo/RT	TG, insulin, glucose, HDL, LDL, HOMA-IR
D’Alonzo et al. (38)	2021	USA	59.3 ± 8.8 / 60.5 ± 8.9	101 (50,51)	RCTs	48w	2s	180 min	NS	NS	Survivors	Insulin, Glucose, HOMA IR
De Luca et al. (39)	2016	Italy	50.2 ± 9.7 / 46.0 ± 2.8	20 (10,10)	RCTs	24w	2s	90min	I-III	Post-m	Survivors, Mastectomy	QoL (FACT-G)
de Paulo et al. (40)	2018	Brazil	63.2 ± 7.1 / 66.6 ± 9.6	36 (18,18)	RCTs	36w	3s	100 min	0–III	Post-m	Survivors, AI Therapy	TG, glucose, HDL, LDL,
Dieli-Conwright et al. (a) (41)	2018	USA	52.8 ± 10.6 / 53.6 ± 10.1	100 (50,50)	RCTs	16w	3s	50-80 min	0–III	Post-m	Survivors, AI Therapy	QoL (FACT-G, SF-36)
Dieli-Conwright et al. (b) (42)	2018	USA	53.6 ± 10.4	100 (50,50)	RCTs	16w	3s	50-80 min	0–III	Pre-m Post-m,	Survivors, Chemo/RT	TG, insulin, glucose, HDL, LDL, SBP, DBP, HOMA-IR
Dieli-Conwright et al. (43)	2019	USA	HBCS 46.8 + 10.2 NHBCS 55.7 + 10.5	100 (50,50)	RCTs	16w	3s	80min	I-III	Post-m	Survivors, Chemo & RT	QoL (FACT-G, SF-36, FACT-B), PF (FACT-G, SF-36), RF, SF (FACT-G, SF-36), CF, EF (FACT-G, SF-36)
Dong et al. (44)	2019	China	48.00 ± 5.54 / 51.63 ± 7.49	60 (30,30)	RCTs	12w	4s	30min	I–III	NS	Undergone surgery	QoL (SF-36), PF, SF, CF, EF
Ergun et al. (65)	2013	Turkey	49.65 ± 8.25 / 50.30 ± 10.37	40 (20,20)	RCTs	12w	3s	75min	NS	Post-m	Survivors, Chemo & RT	QoL (EROTC QLQ-C30), SF
Gal et al. (46)	2021	Netherlands	56.6 ± 9.8 / 58.3 ± 9.5	260 (130, 130)	RCTs	12w	2s	60min	NS	NS	Survivors, Hormonal Tx	QoL (EROTC QLQ-C30), PF, RF, SF, CF, EF
Galiano-Castillo et al. (46)	2016	Spain	47.4 ± 9.6 / 49.2 ± 7.9	81 (40, 41)	RCTs	8w	3s	90min	NS	Pre-m Post-m,	Survivors, Chemo & RT	QoL (EROTC QLQ-C30), PF, RF, SF, CF, EF
Harvie et al. (47)	2019	UK	54.6 ± 11.2/ 55.3 ± 10.5	275 (137,138)	RCTs	48w	2s	70min	Early	Pre-m Post-m,	During treatment	TG, insulin, glucose, HDL, LDL, SBP, DBP, HOMA-IR
Hayes et al. (48)	2013	Australia	51.2 ± 8.8 /53.9 ± 7.7	127 (67,60)	RCTs	32w	3s	45min	0-III	Pre-m Post-m,	During therapy	QoL (FACT-B)
Herrero et al. (49)	2006	Spain	50 ± 5 / 51 ± 10	20 (10,10)	RCTs	8w	3s	90min	I–II	Post-m	Survivors, 2-5 years	QoL (EROTC QLQ-C30), PF
Hojan et al. (50)	2020	Poland	54.4 ± 6.3 / 54.4 ± 6.3	68 (34,34)	RCTs	9w	3s	90min	0–III	NS	During therapy	SBP, DBP
Junghua et al. (51)	2015	Korea	47.1±8.5 / 48.3±8.2	212 (106, 106)	RCTs	4w	3s	80min	0–III	NS	During Rehabilitation Phase	QoL (EROTC QLQ-C30), PF, RF, EF
Kim et al. (52)	2017	Korea	56.0 ± 6.5 / 49.3 ± 4.8	30 (15,15)	RCTs	12w	3s	50min	NS	Post-m	Survivors, Chemo & RT	Insulin
Knobf et al. (53)	2017	USA	51.9 mean	154(76,78)	RCTs	48w	3s	60min	NS	Post-m,Peri,m	Survivors, Non-Endocrine Tx	TG, insulin, HDL, LDL, SBP, DBP, HOMA-IR
Lee et al. (54)	2019	USA	53.5 ± 10.6	100 (50,50)	RCTs	16w	3s	130min	I-III	NS	Survivors, Chemo & RT	LDL
Ligibel et al. (55)	2008	USA	51.2 ± 8.8 / 53.9 ±7.7	101 (51,50)	RCTs	16w	3s	140min	I-III	Post-m	Survivors, Chemo & RT	insulin, glucose, HOMA IR
Milne et al. (56)	2008	Australia	55.2 ± 8.4 / 55.1 ± 8.0	58 (29,29)	RCTs	12w	3s	60min	I–II	NS	Survivors, Chemo & RT	QoL (FACT-G, FACT-B), PF, SF, EF
Mostarda et al. (57)	2017	Brazil	30–59	18 (9,9)	RCTs	4w	3s	70min	I-III	NS	During Chemo/RT	SBP, DBP
Mulero et al. (58)	2008	USA	49.8 ± 6.9/59.6 ± 16.7	21 (12,9)	RCTs	26w	3s	60min	I-III	NS	Survivors, Chemo & RT	QoL (FACT-B)
Mutrie et al. (59)	2007	UK	51.3 ±10.3/51.8 ±8.7	203 (101,102)	RCTs	12w	3s	45min	0–III	NS	During Chemo/RT	QoL (FACT-G), PF, SF, EF
Nuri et al. (60)	2012	Iran	58.27 ± 6.31	29 (14,15)	RCTs	15w	4s	105min	NS	Post-m	Survivors, Chemo & RT	TG, insulin, glucose, HDL, SBP
Paulo et al. (61)	2019	Brazil	63.2 ± 7.1 / 66.6 ± 9.6	36 (18,18)	RCTs	36w	3s	70min	I-III	Post-m	Survivors, AI Therapy	QoL (EROTC QLQ-C30, SF-36), PF (EROTC QLQ-C30, SF-36), RF, SF (EROTC QLQ-C30, SF-36), CF, EF (EROTC QLQ-C30, SF-36)
Schmitz et al. (62)	2005	USA	53.3 ±8.7/ 52.8 ± 7.6	85 (42,43)	RCTs	48w	2s	80min	I-III	Post-m	Survivors, Chemo & RT	insulin, glucose, HOMA IR
Scott et al. (63)	2013	UK	55.6 ±10.2/ 55.9 ± 8.9	90 (47,43)	RCTs	24w	3s	45min	I-III	Post-m,Pre-m,Peri-m	Survivors, Chemo & RT	QoL (FACT-B), HDL, SBP, DBP, HOMA IR
Travier et al. (64)	2015	Netherlands	49.7 ± 8.2/ 49.5 ± 7.9	204 (102,102)	RCTs	18w	2s	60min	NS	Pre-m Post-m,	During Chemo	QoL (EROTC QLQ-C30), PF, RF, SF, CF, EF

N, sample size; CE, combined aerobic with resistance exercise; UC, usual care; D/W, duration per week; S/W, session per week; Min/D, minutes per day; BC, breast cancer; RCTs, randomized controlled trial studies; pre-m, premenopausal; post-m, postmenopausal; per-m, perimenopausal; chemo, chemotherapy; RT, radiotherapy.

### 3.3 Risk of bias and certainty of evidence

**Figure 2:**
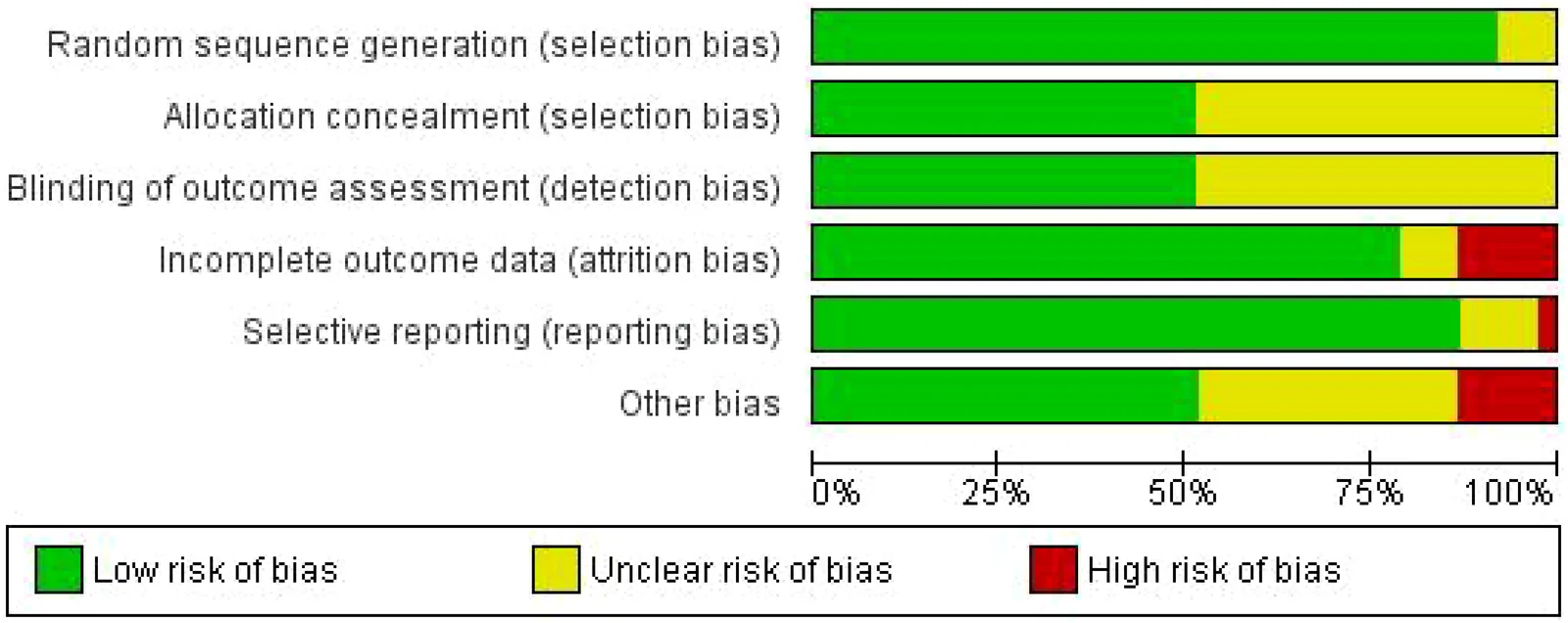
Quality assessment of the included literature.

The risk of bias was assessed using the Cochrane Risk of Bias tool in RevMan 5.4. The quality of the included studies was evaluated across six domains. The blinding of participants and personnel (performance bias) was not assessed, as exercise interventions inherently cannot meet the double-blind eligibility criteria. Although some studies reported single-blind designs, this typically pertained to outcome assessor blinding and does not guarantee the blinding of participants in the intervention groups. For selection bias, most studies demonstrated low risk for random sequence generation, with 34 studies (91.89%) classified as having a low risk; no study reported high risk, and three studies (8.10%) were classified as having an unclear risk. For allocation concealment and detection bias (blinding of outcome assessment), a similar pattern of results was observed: 19 studies (51.35%) were classified as low risk, 18 studies (48.64%) as unclear risk, and no study was classified as high risk. Regarding the attrition domain (incomplete outcome data), 29 studies (78.37%) were classified as low risk, five (13.51%) as high risk, and three (8.10%) as unclear risk. Moreover, for reporting bias (selective reporting), 32 studies (86.48%) were assessed as low risk, one study (2.70%) as high risk, and four studies (10.81%) as unclear risk. Finally, in the domain of other bias, 19 studies (51.35%) were classified as low risk, five (13.51%) as high risk, and 13 (35.13%) as unclear risk (**Figure 2**).

The certainty of evidence for the main outcomes was assessed using the GRADE approach. The certainty of evidence ranged from low to high across outcomes. The main reasons for downgrading the certainty of evidence were substantial inconsistency due to high statistical heterogeneity and imprecision caused by wide confidence intervals crossing the null effect. Detailed GRADE judgments for each outcome are provided in **Supplementary Material S2**.

### 3.4 Meta-analysis

#### 3.4.1 Overall QoL and FACT-B

Pooling different QoL scales allowed estimation of the overall effect on the quality of life of breast cancer patients. The scales that are generally used to assess QoL are EROTC QLQ-C30, FACT-G, MQoL, and SF-36. All of these scales have different methods of assessing QoL, but they are similar in some ways. Some scales use a total score of 100, whereas others use different scoring ranges. Therefore, this meta-analysis used the standardized mean difference (Hedges’ *g*) to account for differences among measurement scales. Twenty-four studies out of 37 met the criteria to conduct this analysis.

**Figure 3:**
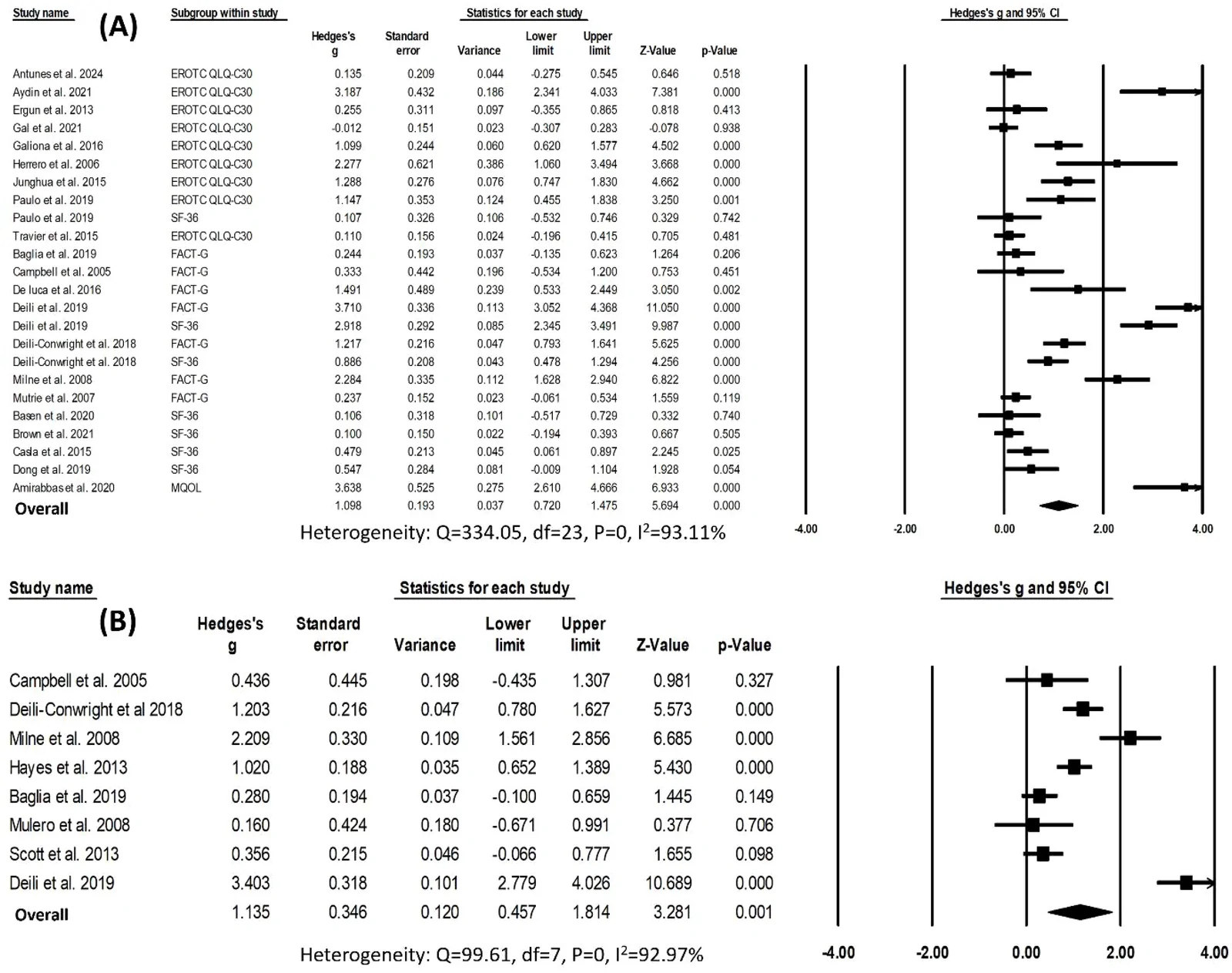
Forest plot of the random-effects analysis showing the effects of combined exercise on **(A)** overall QoL and **(B)** FACT-B.

The positive value of Hedges’ *g* indicates that BC patients who performed combined aerobic and resistance exercise showed significantly greater improvements in overall QoL than those in the usual care group (Hedges’ *g* = 1.10; 95% CI: 0.72, 1.48; *p* < 0.0001) and similarly in FACT B (Hedges’ *g* = 1.14; 95% CI: 0.46, 1.81; *p* = 0.001, **Figure 3**). However, significant heterogeneity indicated variability across the studies (*I*^2^ > 75%) (**Table 2**). To explore the source of heterogeneity and assess the reliability of the results, a sensitivity analysis was conducted by removing each study sequentially from this analysis. Furthermore, the funnel plot and Egger’s test (*p* < 0.0001) showed no significant publication bias for both overall QoL and FACT-B (**Supplementary Material S3**).

**Table 2:** Primary analysis results.

Outcomes	k	N	Hedges' *g* [*95% CI]*	P-value	Heterogeneity			Egger's test (p)
QoL Subscales		CE & UC			Q	I^2^	p	
QoL	24	937,989	1.098 [0.72, 1.475]	<0.0001	334.1	93%	<0.0001	<0.0001
FACT-B	9	416,343	1.135 [0.457, 1.814]	0.001	99.61	93%	<0.0001	NA
PF	19	792,842	0.709 [0.409 - 1.010]	<0.0001	148.2	88%	<0.0001	0.009
RF	11	514,569	0.417 [0.077 - 0.757]	0.016	72.66	86%	<0.0001	0.083
EF	18	824,875	0.414 [0.155 - 0.673]	0.002	114.1	85%	<0.0001	0.078
CF	10	447,498	0.572 [0.166 - 0.978]	0.006	80.52	89%	<0.0001	0.046
SF	16	741,797	0.451 [0.164 -738]	0.002	111.1	87%	<0.0001	0.05
Glucose	8	359,365	-0.221 [-0.57, 0.128]	0.214	33.77	79%	<0.0001	NA
Insulin	9	398, 411	-0.302 [-0.624, 0.020]	0.066	37.69	79%	<0.0001	NA
HOMA	8	432,433	-0.581 [-1.059, 0.103]	0.017	78.21	91%	<0.0001	NA
TG	6	297,304	-0.772 [-1.616, 0.072]	0.073	99.68	95%	<0.0001	NA
HDL	7	340,344	0.828 [ 0.051, 1.606]	0.037	121.5	95%	<0.0001	NA
LDL	6	333,339	-0.644 [-1.466, 0.168]	0.12	111.5	96%	<0.0001	NA
SBP	7	337,325	-0.372 [-0.754, 0.009]	0.056	30.3	80%	<0.0001	NA
DBP	6	323,315	-0.39 [-0.786, -0.008]	0.045	25.82	81%	<0.0001	NA

k, number of studies; N, number of participants; CE, combined aerobic and resistance exercises; UC, usual care; PF, physical functioning; RF, role functioning; EF, emotional functioning; CF, cognitive functioning; SF, social functioning; TG, triglycerides; D/W, duration per week; F, frequency; D/day, duration per day; NA, not applicable.

#### 3.4.2 Subscales of QoL

The results of the subscales of QoL were reported in **Table 1**. Combined aerobic and resistance exercise showed significant moderate to small effects across all subscales compared with usual care. The largest effect was observed for physical functioning (Hedges’ *g* = 0.709; 95% CI: 0.409, 1.010; *p* < 0.0001) and cognitive functioning (Hedges’ *g* = 0.572, 95% CI: 0.166, 0.978; *p* = 0.006), while the smallest effect was observed for role functioning (Hedges’ *g* = 0.417; 95% CI: 0.077 to 0.757; *p* = 0.016). High heterogeneity was observed across all subscales (*I*^2^ > 75%; **Table 2**).

#### 3.4.3 Metabolic blood biomarkers

**Figure 4:**
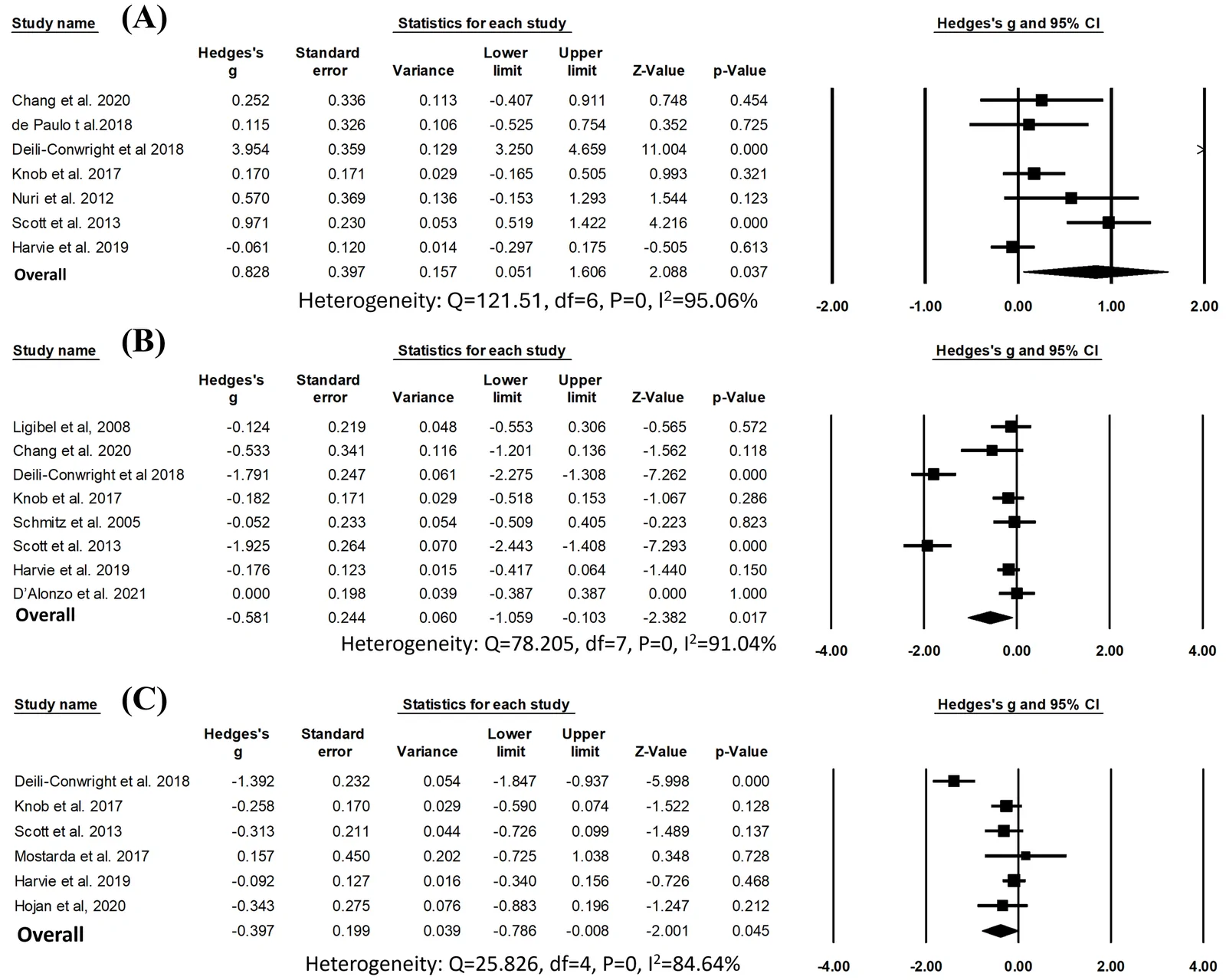
Forest plot of the random-effects analysis showing the effects of combined exercise on **(A)** HDL, **(B)** HOMA-IR, and **(C)** DBP.

Furthermore, metabolic blood biomarkers were analyzed across eligible studies. The meta-analysis indicated that combined aerobic and resistance exercise in breast cancer patients had moderate to low effect sizes for HDL (Hedges’ *g* = 0.828, 95% CI: 0.051, 1.606; p = 0.037), HOMA (Hedges’ *g* = − 0.581; 95% CI: − 1.059, − 0.103; *p* = 0.017) and DBP (Hedges’ *g* = − 0.39; 95% CI: − 0.786, − 0.0008; *p* = 0.045 (**Figure 4**), while other outcomes showed smaller or nonsignificant effect sizes (*p* > 0.05). The heterogeneity across all tests was high, ranging from *I*^2^ = 79% to 95% (*p* < 0.0001; **Table 2**).

### 3.5 Secondary analysis

In the secondary analysis, we examined the effects of intervention duration (duration/week), frequency (sessions/week), and session duration (min/day) of intervention on quality of life and metabolic blood biomarker outcomes.

The exploratory subgroup analyses indicated variations in effect estimates according to intervention duration, exercise frequency, and session duration. Higher effect estimates were observed in some QoL outcomes among studies with longer intervention duration (> 12 weeks), higher frequency (> 3 sessions/week), and longer session duration (> 60 min/session); however, these findings should be interpreted cautiously due to substantial heterogeneity across studies.

The overall quality of life results indicate that combined aerobic and resistance exercise with high frequency (> 3 sessions) and longer session duration (> 60 min) had higher effect sizes (Hedges’ *g* = 1.24, 95% CI: 0.79, 1.69; *p* < 0.0001) and (Hedges’ *g* = 1.29, 95% CI: 0.78, 1.84; *p* < 0.0001), while a shorter intervention duration (≤ 12 weeks) was associated with greater improvement (Hedges’ *g* = 1.51, 95% CI: 0.89, 2.13; *p* < 0.0001; **Table 3**).

**Table 3:** Secondary analysis results of overall QoL and its subscales.

Outcomes	Secondary Analysis		k	N	Hedges' *g* [*95% CI]*	P-value	Heterogeneity	
				CE & UC			I^2^	p
QoL	D/W	≤ 12w	14	555,567	1.51 [0.89, 2.13]	<0.0001	95%	<0.0001
		> 12w	10	382,422	0.53 [0.23, 0.84]	0.0005	75%	<0.0001
	F	≤ 2s	4	208,261	0.33 [-0.06, 0.73]	0.1	73%	0.012
		> 3s	20	697,698	1.24 [0.79, 1.69]	<0.0001	93%	<0.0001
	D/day	≤ 60min	11	530,592	0.85 [0.04, 1.32]	0.0003	92%	<0.0001
		> 60min	13	407,397	1.29 [0.78, 1.84]	<0.0001	92%	<0.0001
FACT-B	D/W	≤ 12w	3	97,87	0.97 [-0.3, 2.24]	0.133	92%	<0.0001
		> 12w	5	223,208	1.23 [0.32, 1.14]	0.008	94%	<0.0001
	D/day	≤ 60min	5	165,147	0.10 [-0.07,0.27]	0.23	85%	<0.0001
		> 60min	3	155,148	1.11 [0.52, 1.70]	<0.0001	92%	<0.0001
PF	D/W	≤ 12w	12	569,635	0.71 [0.33,1.10]	<0.0001	90%	<0.0001
		> 12w	10	338,324	0.90 [0.48, 1.31]	<0.0001	84%	<0.0001
	F	≤ 2s	5	227,279	0.25 [-0.02, 0.52]	0.065	46%	0.115
		> 3s	17	680,680	0.94 [0.58, 1.30]	<0.0001	90%	<0.0001
	D/day	≤ 60min	12	540,601	0.50 [0.20, 0.79]	0.001	82%	<0.0001
		> 60min	10	367,358	1.13 [0.70,1.56]	<0.0001	86%	<0.0001
RF	D/W	≤ 12w	5	206,266	0.19 [-0.17, 0.56]	0.3	72%	0.007
		> 12w	6	308,303	0.60 [0.05, 1.15]	0.032	90%	<0.0001
	F	≤ 2s	3	172,226	0.07 [-0.23, 0.38]	0.641	50%	0.137
		> 3s	8	342,343	0.54 [0.08, 1.0]	0.02	88%	<0.0001
	D/day	≤ 60min	6	328,385	0.04 [-0.12, 0.20]	0.634	11%	0.345
		> 60min	5	186,184	0.84 [0.28, 1.40]	0.003	85%	<0.0001
EF	D/W	≤ 12w	10	408,473	0.32 [0.04, 0.59]	0.025	74%	<0.0001
		> 12w	10	481,469	0.79 [0.21, 1.37]	0.007	94%	<0.0001
	F	≤ 2s	4	217,270	-0.008 [-0.19, 0.17]	0.932	0%	0.99
		> 3s	16	672,672	0.70 [0.31, 1.09]	<0.0001	91%	<0.0001
	D/day	≤ 60min	11	530,592	0.10 [-0.07, 0.27]	0.23	45%	0.052
		> 60min	9	359,350	1.11 [0.15, 1.66]	<0.0001	92%	<0.0001
CF	D/W	≤ 12w	6	245,304	0.48 [0.19, 0.76]	0.001	60%	0.027
		> 12w	4	202,194	0.67 [-0.37, 1.71]	0.209	96%	<0.0001
	F	≤ 2s	3	198,251	0.08 [-0.24, 0.41]	0.617	65%	0.057
		> 3s	7	249,247	0.79 [0.28, 1.31]	0.003	87%	<0.0001
	D/day	≤ 60min	6	293,344	0.30 [-0.004, 0.60]	0.053	70%	0.005
		> 60min	4	154,154	0.94 [0.09, 1.78]	0.03	92%	<0.0001
SF	D/W	≤ 12w	9	375,445	0.28 [0.09, 0.474]	0.004	43%	0.08
		> 12w	10	481,469	0.964 [0.345, 1.584]	0.002	95%	<0.0001
	F	≤ 2s	4	217,270	0.226 [-0.054, 0.507]	0.114	53%	0.094
		> 3s	15	639,644	0.751 [0.321, 1.18]	0.001	93%	<0.0001
	D/day	≤ 60min	10	510,574	0.176 [0.016, 0.337]	0.031	39%	0.098
		> 60min	9	346,340	0.991 [0.826, 1.156]	<0.0001	94%	<0.0001

k, number of studies; N, number of participants; D/W, duration per week; F, frequency; D/day, duration per day; CE, combined aerobic with resistance exercise; UC, usual care.

Similar findings were observed for FACT-B scores, with higher effect sizes for longer intervention duration (> 12 weeks) and longer session duration (> 60 min). Furthermore, the quality of life subscales showed significant effects for PF, RF, SF, CF, and EF with longer intervention duration (> 12 weeks), higher frequency (> 3 sessions), and longer session duration (> 60 min/day), with Hedges’ *g* ranging from 0.54 to 1.13 (*p* < 0.05), whereas shorter intervention duration, low frequency, and shorter session duration (≤ 60 min) showed smaller or nonsignificant effects (Hedges’ *g* = − 0.008–0.33, *p* > 0.05). Heterogeneity ranged from 35% to 95%, indicating moderate to high heterogeneity across studies (**Table 3**).

On the other hand, the metabolic blood marker analysis indicated that a shorter intervention duration (≤ 12 weeks) and high exercise frequency (> 3 sessions/week) had a significant effect on reducing HOMA-IR (*p* < 0.05), indicating improved insulin sensitivity. Moreover, SBP and DBP decreased considerably when session duration was ≤ 60 min/day. In contrast, fasting glucose and lipid profiles (triglycerides, HDL, and LDL) showed no statistically significant effects (*p* > 0.05).

Secondary analyses were performed to explore the influence of exercise characteristics, including intervention duration, frequency, and session duration, on the observed outcomes. Although heterogeneity was generally high across analyses, some analyses showed low to negligible heterogeneity (*I*^2^ = 0-29%; **Table 4**). Please refer to **Supplementary Material S3** for graphical representations of all forest and funnel plots for the primary and secondary analyses.

**Table 4:** Secondary analysis results of metabolic blood markers.

Outcomes	Secondary Analysis		K	N	Hedges' *g* [*95% CI]*	P-value	Heterogeneity	
				CE & UC			I^2^	p
Glucose	D/W	≤ 12w	3	172,173	-0.07 [-0.28, 0.14]	0.505	0%	0.612
		> 12w	5	187,192	-0.26 [-0.85, 0.33]	0.382	87%	<0.0001
	F	≤ 2s	3	91,96	-0.21 [-0.56, 0.14]	0.24	29%	0.245
		> 3s	5	268,269	-0.21 [-0.75, 0.32]	0.429	87%	<0.0001
Insulin	D/W	≤ 12w	3	145,148	-0.24 [-0.47, -0.01]	0.038	0%	0.521
		> 12w	6	253,263	-0.282 [-0.77, 0.20]	0.252	86%	<0.0001
	F	≤ 2s	3	91,97	-0.007 [-0.461, 0.446]	0.830	57%	0.097
		> 3s	6	307,314	-0.438 [-0.852, -0.024]	0.038	83%	<0.0001
	D/day	≤ 60min	4	211,218	-0.17 [-0.36, 0.02]	0.074	0%	0.487
		> 60min	5	187,193	-0.34 [-0.95, 0.27]	0.275	88%	<0.0001
HOMA IR	D/W	≤ 12w	3	329,334	-0.21 [-0.39, -0.02]	0.031	0%	0.608
		> 12w	5	327,329	-0.77 [-1.60, -0.06]	0.069	94%	<0.0001
	D/day	≤ 60min	4	260,259	-0.68 [-1.39, 0.02]	0.058	92%	<0.0001
		> 60min	4	172,174	-0.48 [-1.28, 0.31]	0.230	92%	<0.0001
TG	D/W	≤ 12w	3	172,173	-0.078 [-0.29, 0.13]	0.469	0%	0.556
		> 12w	3	125,131	-1.45 [-3.44, 0.539]	0.153	97%	<0.0001
	D/day	≤ 60min	3	219,226	-0.19 [-0.40, 0.02]	0.082	14%	0.315
		> 60min	3	78,78	-1.31 [-3.61, 0.98]	0.263	97%	<0.0001
HDL	D/W	≤ 12w	3	172,173	-0.01 [-0.22, 0.20]	0.924	0%	0.93
		> 12w	4	168,171	1.40 [-1.83, 2.81]	0.053	97%	<0.0001
	D/day	≤ 60min	4	262,266	0.31 [-0.13, 0.75]	0.167	81%	0.001
		> 60min	3	78,78	1.54 [-0.82, 3.91]	0.201	97%	<0.0001
LDL	D/W	≤ 12w	3	172,173	-0.04 [-0.25, 0.17]	0.719	0%	0.9
		> 12w	3	161,166	-1.31 [-3.07, 0.44]	0.143	98%	<0.0001
SBP	D/W	≤ 12w	3	163,150	-0.21 [-0.433, 0.009]	0.060	0%	0.7
		> 12w	4	174,175	-0.51 [-1.19, 0.17]	0.143	89%	<0.0001
	D/day	≤ 60min	3	242,235	-0.20 [-0.38, -0.02]	0.031	0%	0.897
		> 60min	4	95,90	-0.51 [-1.34, 0.31	0.223	85%	<0.0001
DBP	D/W	≤ 12w	3	163, 155	-0.12 [-0.34, 0.10]	0.29	0%	0.58
		> 12w	3	160,160	-0.64 [-1.32, 0.04]	0.063	89	<0.0001
	D/day	≤ 60min	3	242,235	-0.182 [-0.361, 0.003]	0.046	0%	0.579
		> 60min	3	81,80	-0.578 [-1.485, 0.329]	0.212	85%	0.001

k, number of studies; N, number of participants; D/W, duration per week; F, frequency; D/day, duration per day.

### 3.6 Exploration of sources of heterogeneity through subgroup analyses

Subgroup analyses were conducted to further evaluate sources of heterogeneity between studies. Subgroup analyses were conducted based on exercise supervision status, treatment status, and QoL measurement scales.

Subgroup analyses related to exercise supervision status showed considerable heterogeneity for both supervised and hybrid/home-based exercise interventions. Supervised exercise had high heterogeneity for metabolic outcomes (*I*^2^ = 89.92%–97.86%) and QoL analysis (*I*^2^ = 94.18%). Similarly, substantial heterogeneity was noted in hybrid/home-based interventions, especially for QoL outcomes (*I*^2^ = 89.64%), whereas some metabolic outcomes had lower heterogeneity (*I*^2^ = 0%–66.59%).

Subgroup analyses based on treatment status demonstrated significant improvements in overall QoL and several QoL subscales for both active-treatment and posttreatment groups, with larger effect estimates generally observed in post-treatment participants. However, heterogeneity was much higher in most of the posttreatment analyses (*I*^2^ > 88%), whereas active-treatment subgroups showed lower or moderate heterogeneity in several outcomes. Among metabolic outcomes, treatment-status subgroups had varying, mostly nonsignificant effects on blood pressure outcomes.

In the overall QoL analysis, the results indicate significant improvements in favor of the intervention group, while greater improvements were observed on the Fact-G scale results, suggesting that incorporating combined aerobic and resistance exercise in breast cancer patients could provide benefits.

Among QoL subscales, large to moderate effect sizes were observed in the SF and EF (Fact-G) and CF (SF-36; *p* < 0.05). Surprisingly, the SF-36 group showed nonsignificant results across all QoL subscales except CF. The heterogeneity across studies was moderate to high (> 50%); some subscales showed low heterogeneity. A graphical representation of the analysis is provided in **Supplementary Material S3**. Furthermore, dose-related secondary analyses were considered exploratory and were interpreted cautiously.

Menopausal status could not be examined through subgroup analysis because no study exclusively included premenopausal participants, while several studies included mixed menopausal populations or did not report menopausal status.

## 4 Discussion

The present meta-analysis provides a comprehensive assessment of CE effects on QoL and metabolic blood biomarker outcomes in BC patients. It includes a diverse range of studies, encompassing different populations, countries, ages, menopausal statuses, cancer types, and exercise doses. This broad inclusion increases the scope of the review and provides a comprehensive interpretation of the results, while also introducing substantial clinical heterogeneity.

### 4.1 QoL and its subscales

Specifically, we found that CE significantly improved QoL and exerted significant effects (*p* < 0.05) on some outcomes related to metabolic blood biomarkers of BC patients. The findings suggest that CE may represent a beneficial nonpharmacological intervention for improving overall QoL and multidimensional QoL outcomes among women with breast cancer.

These findings align with previous meta-analyses, which found that CE was particularly effective in enhancing QoL, alleviating depression, and mitigating anxiety (66, 67). Physiologically, aerobic exercises help improve cardiorespiratory function and cardiovascular health (17, 65), whereas resistance exercise improves physical function, lean muscle mass, and muscle strength (68). Therefore, the combination of aerobic and resistance interventions had significant effects on QoL outcomes.

Similar results were observed for the multidimensional QoL subscales, indicating a significant improvement with small to moderate effect sizes across all subscales, including PF, RF, EF, CF, and SF (69). Previous studies have found a positive association between exercise in BC patients, with beneficial effects on PF (17) and CF (70), potentially through reductions in oxidative stress and hormonal stimulation (71).

The mechanisms underlying these improvements are multifactorial. These mechanisms include dopamine and norepinephrine, alongside neurotrophic factors such as brain-derived neurotrophic factor (BDNF), which strengthen neural integrity and enhance neuronal growth and connectivity (72, 73). In addition, both preclinical and clinical studies suggest that regular exercise can stimulate structural adaptations in the brain, particularly in the prefrontal cortex and hippocampal regions (74, 75), alongside increasing cerebral circulation and angiogenesis, promoting the transport of oxygen and metabolic substrates, and improving brain function and cognitive ability (76). Regular exercise contributes to metabolic regulation by stabilizing blood sugar and hormone levels (77), and reduces chronic inflammation and alleviates oxidative stress (78), which are key contributors to enhanced emotional and cognitive function.

In addition, chemotherapy and other treatments for breast cancer patients can induce hypothalamic–pituitary–adrenal (HPA) axis dysregulation, which may contribute to endocrine dysfunction (79) and negatively impact QoL (80, 81). CE may help mitigate some of these adverse effects by improving physiological functioning and psychological well-being, gradually restoring normal social function, improving independence in daily living, and enhancing QoL.

A meta-analysis conducted by Lipsett et al. (2017) showed that breast cancer patients participating in aerobic, resistance, or CE during adjuvant radiotherapy experienced improvements in fatigue and QoL (67). These findings reinforce the interpretation that BC patients should engage in CE exercise during and after treatment to mitigate potential risks, including reduced QoL and physical, role, emotional, cognitive, and social functioning.

However, the magnitude of improvement in quality-of-life domains may be dependent on the attributes of the exercise intervention. Perhaps the aerobic and resistance components may affect different aspects of recovery. Aerobic exercise may mainly contribute to cardiovascular fitness and metabolic regulation, whereas resistance exercise may offer added benefits via improvements in muscle strength, physical function, and maintenance of lean tissue. The combined approach may therefore provide a broader rehabilitation stimulus by addressing both physiological and functional limitations commonly experienced by breast cancer patients.

### 4.2 Metabolic blood biomarkers

Our meta-analysis suggests that CE may improve HOMA-IR, HDL, and DBP outcomes among women with breast cancer. These findings align with prior research, indicating that breast cancer patients could yield metabolic benefits by incorporating exercise into their lifestyle (21, 80, 82). Although nonsignificant results were observed for glucose, insulin sensitivity, triglycerides, LDL, and SBP, a small to moderate favorable effect size was identified across these outcomes.

Increased glucose and insulin levels are associated with BC cell growth and recurrence (83). Exercise reduces glucose levels, potentially disrupting tumor metabolism and the Warburg effect, an altered metabolic pathway in cancer cells that uses rapid energy production through aerobic glycolysis instead of the more efficient method (oxidative phosphorylation) (84). Previous studies indicate that CE is helpful in lowering insulin-like growth factor (IGF-1) (80), which strengthens the cancer growth signal and prevents apoptosis. Furthermore, resistance training improves muscle strength and glycogen synthesis (85), while aerobic exercise improves mitochondrial performance and glucose metabolism (86). In combination, they enhance glycemic regulation by decreasing insulin resistance and optimizing glucose metabolism (87).

For triglycerides, our meta-analysis results demonstrate a small to moderate effect size reduction favoring the CE group, although the effect was not statistically significant (*p* > 0.073). Previous studies demonstrated that CE exercise is more efficient at improving lipid profiles (21, 82), whereas other studies have disputed this, stating that other exercises (e.g., high-intensity training [HIT]) may be comparatively more effective in improving triglyceride levels (88). A recent study demonstrated the modest impact of exercise on triglycerides (80), consistent with our results.

Regarding cholesterol outcomes, this analysis revealed a significant improvement in HDL levels, whereas no statistically significant effect was identified for LDL. A recent network meta-analysis demonstrated that CE was the most effective exercise intervention for increasing HDL cholesterol in breast cancer patients, with significant effects compared with other exercise interventions (80). Moreover, elevated HDL is inversely correlated with the risk of BC progression, influencing inflammatory properties, low estrogen levels, and absolute mammographic density (89, 90). Although studies regarding the impact of CE on LDL cholesterol remain inconsistent, substantial heterogeneity may have contributed to these inconsistencies.

Furthermore, we compared blood pressure (SBP and DBP) between the CE and UC groups. The analysis demonstrated beneficial effects on DBP, while SBP showed a nonstatistically significant effect. Exercise improves endothelial function, reduces arterial stiffness, and increases peripheral blood flow through increased nitric oxide, thereby improving blood pressure regulation (91). The inconsistencies in our results could be attributed to the high heterogeneity (*I*^2^ > 75%) across the included studies, suggesting substantial differences between the included studies.

The current findings indicate that the metabolic response to combined exercise may not be comparable across all biomarkers. The observed improvements in HOMA-IR, HDL, and DBP suggest potential metabolic benefits; the nonsubstantial effects observed for several other biomarkers demonstrate the complexity of exercise-induced physiological adaptations in breast cancer populations. The differences in metabolic response among individuals may be due to variations in baseline metabolic status, treatment exposure, and the intensity and duration of the exercise intervention.

### 4.3 Secondary analysis

In this meta-analysis, we also performed a secondary analysis to further identify the sources of heterogeneity and assess the reliability of the interpretations across the included studies. The secondary evaluation was conducted based on exercise dose characteristics, such as duration per week, frequency (sessions/week), and session duration (min/day).

Our analysis demonstrated significant effects of CE with respect to exercise dose in BC patients. It showed that CE with a longer intervention duration, higher frequency, and longer session duration significantly improved the overall QoL and its subscales. In a previous study, long-term exercise was shown to mitigate BC symptoms related to QoL, suggesting that it is a safe, feasible, and effective approach to recovery that enhances physical and mental outcomes. Lin et al. (2023) further demonstrated that a 6-month exercise program resulted in greater improvements in QoL (92). These results are consistent with other research that found that a longer duration (> 12 weeks), high frequency (> 3 sessions), and session duration (> 60 min) are more effective in improving all types of QoL for BC patients (17, 22, 93). In addition, after breast cancer chemotherapy and radiotherapy, there may be changes in musculoskeletal metabolism that cannot be reversed with short-term exercise (94, 95), indicating that longer exercise duration, higher frequency, and longer session duration may be more effective in mitigating these risks.

The interpretations for metabolic blood biomarkers were not consistent with the QoL results. They showed that both shorter and longer exercise duration and frequency had different effects on different MBB outcomes. Our secondary analysis indicated that a shorter duration per week (≤ 12 weeks) and a high-frequency approach (> 3 sessions) of CE were associated with lower HOMA-IR, suggesting improved insulin resistance. This is consistent with recent meta-analyses that have shown that CE has a synergistic effect, combining improved glucose disposal in resistance-trained muscle with better insulin signaling from aerobic activity (80). This study indicates that metabolic “remodeling” of the lipid profile may necessitate a greater cumulative dose or extended intervention durations compared to those necessary to enhance insulin sensitivity.

While insulin sensitivity and HOMA-IR show significant improvement, the results for glucose and lipid profiles (HDL, LDL) are insignificant (*p* > 0.05) across all exercise doses. Specifically, fasting glucose, triglycerides, HDL, and LDL show a large effect with a longer duration per week (> 12 weeks) compared with a shorter duration (≤ 12 weeks), consistent with studies showing that long-term exercise is effective at reducing these risks in BC patients (96, 97).

Moreover, a notable decrease in SBP and DBP was observed following the short exercise dose session duration (≤ 60 min) compared with longer sessions (> 60 min), indicating that regular exercise sessions may be most effective for controlling treatment-induced hypertension while avoiding the systemic tiredness often observed in oncology populations during long-term sessions (98).

The secondary analyses categorized by the exercise characteristics provided exploratory information about possible heterogeneity of intervention effects. However, the results should be interpreted with caution, as the cut-off values were based on the distribution of included studies and the availability of subgroups rather than established dose–response thresholds. Therefore, these analyses cannot determine an optimal exercise dose.

### 4.4 Potential sources of heterogeneity and subgroup findings

Substantial heterogeneity was found in several outcomes in the current meta-analysis. Subgroup analyses were performed according to exercise supervision status, treatment status, and QoL measurement instruments to explore potential causes of this variability. The results suggested that the treatment phase and characteristics of intervention delivery may explain differences in exercise responses, but substantial heterogeneity remained in most subgroups, indicating that a variety of clinical and methodological factors contribute to the observed effects.

Although posttreatment participants generally showed greater improvements in several QoL domains compared with active-treatment participants, these findings should be interpreted cautiously because subgroup analyses were exploratory and may reflect differences in participant characteristics and intervention protocols.

Variability in observed effects could be attributed to the inclusion of various exercise delivery methods and clinical settings. The intervention settings in the selected studies were diverse, including supervised, home-based, and hybrid exercise programs, which may have differed in exercise adherence, monitoring, intensity control, and intervention integrity. Furthermore, participants varied in treatment status, including women undergoing active treatment and breast cancer survivors after treatment completion. These differences may affect physical capacity, treatment-related symptoms, metabolic status, and response to exercise interventions. Subgroup analyses by treatment status and exercise supervision indicated remaining heterogeneity, suggesting that intervention settings and clinical characteristics may contribute to differences in exercise outcomes.

### 4.5 Strengths and limitations

This analysis provides a comprehensive view of the effects of CE on metabolic blood markers (glucose, insulin, HOMA-IR, TG, HDL, LDL, SBP, and DBP) and overall QoL and its subscales (PF, RF, EF, CF, and SF). Additionally, we performed secondary and subgroup analyses to further investigate the source of heterogeneity and the reliability of the interpretation by taking into account the exercise dose (intervention duration, frequency, and session duration) and individual scales (EROTC QLQ-C30, FACT-G, SF-36, and MQoL), which have not been examined in previous research (20, 22, 66). Moreover, this analysis included a relatively large number of studies (*n* = 37), particularly for QoL (*n* = 24), enhancing the statistical power and precision of our interpretations. Furthermore, where possible, the funnel plot, Egger’s test, sensitivity analysis, and trim-and-fill analysis were performed to explore sources of heterogeneity and enhance the credibility and integrity of the findings. These findings may help clinicians, rehabilitators, and caregivers address these risk factors by incorporating CE into individualized exercise recommendations and encouraging its implementation to mitigate further complications.

However, this analysis also has some limitations. First, although a substantial body of studies was included, evidence was limited for specific outcomes such as QoL subscales (*n* = 10–19) and even more limited for MBB outcomes (*n* = 6–9); this limited evidence may reduce the reliability and interpretability of the corresponding pooled estimates. Additionally, the evidence base was limited for some outcomes; there were insufficient studies to conduct the funnel plot, and Egger’s test and trim-and-fill analysis were not possible to conduct, which may also have contributed to the heterogeneous results. Secondly, in the secondary analysis, variability in exercise interventions may further affect the generalizability of these findings.

Additionally, differences in exercise protocols, participant characteristics, treatment status, and outcome measurement instruments may have contributed to the observed heterogeneity. Although subgroup analyses were conducted to explore these factors, residual heterogeneity remained. Similarly, subgroup analyses by menopausal status and cancer stage were not possible due to the lack of reporting and category-specific data in the included studies. Another limitation is the clinical and methodological heterogeneity of the interventions included. Heterogeneity in the mode of exercise delivery, level of supervision, treatment phase, and survivorship status may limit the generalizability of pooled estimates.

Beyond the methodological limitations of the included studies, practical challenges related to exercise implementation among breast cancer survivors need to be taken into consideration. Exercise participation may be affected by treatment-related symptoms, fatigue, reduced physical capacity, psychological barriers, and differences in individual readiness for exercise. Moreover, the type of exercise program (supervised vs. home-based vs. hybrid) may influence adherence, monitoring, and consistency of the intervention. These considerations may limit the effectiveness of implementing comparable exercise prescriptions for all breast cancer survivors and emphasize the importance of tailored exercise planning. Further research is required to address these constraints and provide guidance for clinicians, caregivers, and other stakeholders on implementing CE according to individual abilities.

### 4.6 Clinical and practical implications

A large number of systematic reviews and network meta-analyses have established a generalized benefit of physical activity for oncological populations. However, the current clinical literature often combines different exercise modalities or fails to provide a detailed evaluation of dose–response parameters required for clinical practice. This review specifically separates itself from existing evidence by exclusively isolating and quantifying the unique effects of CE in female breast cancer cohorts.

Unlike previous reviews that pooled heterogeneous cancer populations or mixed exercise interventions, this study exclusively included randomized controlled trials evaluating CE and also addresses significant structural gaps in our understanding of how intervention duration, frequency, and individual session duration affect physiological and psychosocial outcomes. Additionally, this research simultaneously examines multidimensional quality-of-life subscales with metabolic blood biomarkers, which provides a more holistic interpretation of psychosocial and metabolic recovery outcomes.

This review shows that CE could have beneficial effects on general quality of life, various QoL subdomains, and metabolic blood biomarkers. Secondary analyses further suggest that exercise dose characteristics may influence the extent and nature of adaptations noted in breast cancer patients, emphasizing the necessity of tailored and outcome-specific exercise programming in oncology rehabilitation settings. However, these findings should be considered with caution because substantial heterogeneity was identified in numerous analyses, and the majority of the metabolic outcomes were based on a small number of studies.

From a clinical perspective, the present findings suggest that CE may be considered a supportive adjunctive intervention in breast cancer rehabilitation. However, further larger and higher‐quality randomized controlled trials are needed to optimize exercise recommendations, define appropriate dose–response relationships, and characterize the long‐term metabolic and psychological adaptations of CE in breast cancer populations.

From a practical point of view, the results of this meta-analysis provide important considerations for clinicians, rehabilitation professionals, and researchers working in breast cancer care. Aerobic and resistance exercise combined had beneficial effects on quality of life and some metabolic outcomes, but the interpretation of these results should take into account individual patient characteristics and clinical circumstances. The variability noted across interventions indicates that exercise programs should not be applied as a universal protocol, but rather tailored to treatment status, functional capacity, exercise tolerance, and rehabilitation goals. Therefore, the current results suggest the need for individualized exercise strategies in breast cancer rehabilitation programs.

## 5 Conclusion

The quantitative interpretations of this meta-analysis demonstrated that CE exercise was associated with improvements in overall QoL and its subscales, with favorable effects observed for selected metabolic blood biomarkers. Although several metabolic-related outcomes did not reach statistical significance, the findings suggest variability in metabolic responses among BC patients. Furthermore, exploratory secondary analyses suggested that CE exercise characteristics, including exercise duration, frequency, and session duration, may influence the magnitude of effects on metabolic-related outcomes and overall QoL and its subscales; however, these findings require cautious interpretation due to substantial heterogeneity. These findings support the consideration of CE as a supportive component of exercise-based rehabilitation programs for BC patients, while recognizing the need for individualized exercise prescription. Furthermore, CE may provide an accessible supportive approach to improving health-related outcomes during and after breast cancer treatment. CE may be considered by clinicians and caregivers as an adjuvant intervention to mitigate metabolic-related risks and improve QoL. Further research is required to clarify the effects of CE on metabolic blood biomarkers and establish more conclusive evidence.

## Data Availability

The raw data supporting the conclusions of this article will be made available by the authors, without undue reservation.
