# Supplementary material for: The effects of combined aerobic and resistance exercise on metabolic blood biomarkers and quality of life in patients with breast cancer: a systematic review meta-analysis
Source: Front Oncol. 2026 Sep 11;16:1900945. doi: 10.3389/fonc.2026.1900945 (PMC13612194; doi:10.3389/fonc.2026.1900945)
Supplement: Supplementary file 1 [file DataSheet1.docx]

**The effects of combined aerobic and resistance exercise on metabolic blood biomarkers and quality of life in breast cancer patients: a systematic review and meta-analysis**

**Search Strategy**

1. **PubMed**

| 1 | "exercise"[MeSH Terms] OR "physical exercise"[Title/Abstract] OR "exercise"[Title/Abstract] OR "combined exercise"[Title/Abstract] OR "combined training"[Title/Abstract] OR "combined aerobic resistance exercise"[Title/Abstract] OR ("aerobic"[Title/Abstract] AND "resistance exercise"[Title/Abstract]) OR "aerobic exercise"[Title/Abstract] OR "resistance exercise"[Title/Abstract] OR "resistance training"[Title/Abstract] OR "supervised training"[Title/Abstract] OR "supervised exercise"[Title/Abstract] OR "interventional training"[Title/Abstract] OR "interventional exercise"[Title/Abstract] OR "strength training"[Title/Abstract] OR "exercise training"[Title/Abstract] OR ("aerobic"[Title/Abstract] AND "strength training"[Title/Abstract]) OR ("aerobic"[Title/Abstract] AND "strength exercise"[Title/Abstract]) |
| --- | --- |
| 2 | "breast neoplasms"[MeSH Terms] OR "breast neoplasms"[MeSH Terms] OR "breast neoplasms"[MeSH Terms] OR "neoplasm breast"[Title/Abstract] OR "breast tumor"[Title/Abstract] OR "breast tumors"[Title/Abstract] OR "breast carcinoma"[Title/Abstract] OR "patient"[Title/Abstract] OR "female"[Title/Abstract] OR "survivor"[Title/Abstract] |
| 3 | "metabolic syndrome"[MeSH Terms] OR "metabolic biomarker"[Title/Abstract] OR "metabolic marker"[Title/Abstract] OR "metabolic markers"[Title/Abstract] OR "metabolic syndrome"[Title/Abstract] |
| 4 | "lipase"[MeSH Terms] OR "triglyceride"[Title/Abstract] OR "triglycerides"[Title/Abstract] OR "serum triglycerides"[Title/Abstract] OR "Triacylglycerols"[Title/Abstract] OR "Triacylglycerol"[Title/Abstract] |
| 5 | "cholesterol"[MeSH Terms] OR "HDL-Cholesterol"[Title/Abstract] OR "LDL-Cholesterol"[Title/Abstract] OR "HDL-Cholesterol"[Title/Abstract] OR "LDL-Cholesterol"[Title/Abstract] OR "high density lipoprotein"[Title/Abstract] OR "low density lipoprotein"[Title/Abstract] OR "lipid profile"[Title/Abstract] OR "cholesterol levels"[Title/Abstract] OR "Epicholesterol"[Title/Abstract] |
| 6 | (("fasted"[All Fields] OR "fasting"[MeSH Terms] OR "fasting"[All Fields] OR "fastings"[All Fields] OR "fasts"[All Fields]) AND "blood glucose"[MeSH Terms]) OR "fasting glucose"[Title/Abstract] OR "blood glucose levels"[Title/Abstract] OR "glucose metabolism"[Title/Abstract] |
| 7 | (("fasted"[All Fields] OR "fasting"[MeSH Terms] OR "fasting"[All Fields] OR "fastings"[All Fields] OR "fasts"[All Fields]) AND "insulin"[MeSH Terms]) OR "insulin levels"[Title/Abstract] OR "fasting serum insulin"[Title/Abstract] OR "insulin resistance"[Title/Abstract] OR "insulin sensitivity"[Title/Abstract] |
| 8 | "blood pressure"[MeSH Terms] OR "blood pressure determination"[MeSH Terms] OR "arterial pressure"[MeSH Terms] OR "systolic blood pressure"[Title/Abstract] OR "diastolic blood pressure"[Title/Abstract] OR "hypertension"[Title/Abstract] OR "high blood pressure"[Title/Abstract] OR "stroke"[Title/Abstract] OR "sbp"[Title/Abstract] OR "sbp"[Title/Abstract] OR "systolic bp"[Title/Abstract] OR "DBP"[Title/Abstract] OR "diastolic bp"[Title/Abstract] |
| 9 | "quality of life"[MeSH Terms] OR "health related quality of life"[Title/Abstract] OR "QoL"[Title/Abstract] OR "well-being"[Title/Abstract] OR "life satisfaction"[Title/Abstract] OR "functional status"[Title/Abstract] OR "SF-36"[Title/Abstract] OR "eortc qlq c30"[Title/Abstract] OR "WHOQOL"[Title/Abstract] OR "Fact-B"[Title/Abstract] OR "Fact-G"[Title/Abstract] OR "physical functioning"[Title/Abstract] OR "emotional functioning"[Title/Abstract] OR "social functioning"[Title/Abstract] OR "role functioning"[Title/Abstract] OR "cognitive functioning"[Title/Abstract] OR "global health status"[Title/Abstract] OR "mental health"[Title/Abstract] OR "bodily pain"[Title/Abstract] OR "psychological well bieng"[Title/Abstract] |
| 10 | (((((((((((((("exercise"[MeSH Terms] OR "physical exercise"[Title/Abstract] OR "exercise"[Title/Abstract] OR "combined exercise"[Title/Abstract] OR "combined training"[Title/Abstract] OR "combined aerobic resistance exercise"[Title/Abstract] OR ("aerobic"[Title/Abstract] AND "resistance exercise"[Title/Abstract]) OR "aerobic exercise"[Title/Abstract] OR "resistance exercise"[Title/Abstract] OR "resistance training"[Title/Abstract] OR "supervised training"[Title/Abstract] OR "supervised exercise"[Title/Abstract] OR "interventional training"[Title/Abstract] OR "interventional exercise"[Title/Abstract] OR "strength training"[Title/Abstract] OR "exercise training"[Title/Abstract] OR ("aerobic"[Title/Abstract] OR "strength training"[Title/Abstract]) OR ("aerobic"[Title/Abstract] OR "strength exercise"[Title/Abstract])) AND ("breast neoplasms"[MeSH Terms] OR "breast neoplasms"[MeSH Terms] OR "breast neoplasms"[MeSH Terms] OR "neoplasm breast"[Title/Abstract] OR "breast tumor"[Title/Abstract] OR "breast tumors"[Title/Abstract] OR "breast carcinoma"[Title/Abstract] OR "patient"[Title/Abstract] OR "female"[Title/Abstract])) OR "survivor"[Title/Abstract]) AND ("metabolic syndrome"[MeSH Terms] OR "metabolic biomarker"[Title/Abstract] OR "metabolic marker"[Title/Abstract] OR "metabolic markers"[Title/Abstract])) OR "metabolic syndrome"[Title/Abstract]) AND ("lipase"[MeSH Terms] OR "triglyceride"[Title/Abstract] OR "triglycerides"[Title/Abstract] OR "serum triglycerides"[Title/Abstract] OR "Triacylglycerols"[Title/Abstract])) OR "Triacylglycerol"[Title/Abstract]) AND ("cholesterol"[MeSH Terms] OR "HDL-Cholesterol"[Title/Abstract] OR "LDL-Cholesterol"[Title/Abstract] OR "HDL-Cholesterol"[Title/Abstract] OR "LDL-Cholesterol"[Title/Abstract] OR "high density lipoprotein"[Title/Abstract] OR "low density lipoprotein"[Title/Abstract] OR "lipid profile"[Title/Abstract] OR "cholesterol levels"[Title/Abstract])) OR "Epicholesterol"[Title/Abstract]) AND ((("fasted"[All Fields] OR "fasting"[MeSH Terms] OR "fasting"[All Fields] OR "fastings"[All Fields] OR "fasts"[All Fields]) AND "blood glucose"[MeSH Terms]) OR "fasting glucose"[Title/Abstract] OR "blood glucose levels"[Title/Abstract])) OR "glucose metabolism"[Title/Abstract]) AND ((("fasted"[All Fields] OR "fasting"[MeSH Terms] OR "fasting"[All Fields] OR "fastings"[All Fields] OR "fasts"[All Fields]) AND "insulin"[MeSH Terms]) OR "insulin levels"[Title/Abstract] OR "fasting serum insulin"[Title/Abstract] OR "insulin resistance"[Title/Abstract])) OR "insulin sensitivity"[Title/Abstract]) AND ("blood pressure"[MeSH Terms] OR "blood pressure determination"[MeSH Terms] OR "arterial pressure"[MeSH Terms] OR "systolic blood pressure"[Title/Abstract] OR "diastolic blood pressure"[Title/Abstract] OR "hypertension"[Title/Abstract] OR "high blood pressure"[Title/Abstract] OR "stroke"[Title/Abstract] OR "sbp"[Title/Abstract] OR "sbp"[Title/Abstract] OR "systolic bp"[Title/Abstract] OR "DBP"[Title/Abstract])) OR "diastolic bp"[Title/Abstract]  (((((((((((((((("exercise"[MeSH Terms] OR "physical exercise"[Title/Abstract] OR "exercise"[Title/Abstract] OR "combined exercise"[Title/Abstract] OR "combined training"[Title/Abstract] OR "combined aerobic resistance exercise"[Title/Abstract] OR ("aerobic"[Title/Abstract] AND "resistance exercise"[Title/Abstract]) OR "aerobic exercise"[Title/Abstract] OR "resistance exercise"[Title/Abstract] OR "resistance training"[Title/Abstract] OR "supervised training"[Title/Abstract] OR "supervised exercise"[Title/Abstract] OR "interventional training"[Title/Abstract] OR "interventional exercise"[Title/Abstract] OR "strength training"[Title/Abstract] OR "exercise training"[Title/Abstract] OR ("aerobic"[Title/Abstract] OR "strength training"[Title/Abstract]) OR ("aerobic"[Title/Abstract] OR "strength exercise"[Title/Abstract])) AND ("breast neoplasms"[MeSH Terms] OR "breast neoplasms"[MeSH Terms] OR "breast neoplasms"[MeSH Terms] OR "neoplasm breast"[Title/Abstract] OR "breast tumor"[Title/Abstract] OR "breast tumors"[Title/Abstract] OR "breast carcinoma"[Title/Abstract] OR "patient"[Title/Abstract] OR "female"[Title/Abstract])) OR "survivor"[Title/Abstract]) AND ("metabolic syndrome"[MeSH Terms] OR "metabolic biomarker"[Title/Abstract] OR "metabolic marker"[Title/Abstract] OR "metabolic markers"[Title/Abstract])) OR "metabolic syndrome"[Title/Abstract]) AND ("lipase"[MeSH Terms] OR "triglyceride"[Title/Abstract] OR "triglycerides"[Title/Abstract] OR "serum triglycerides"[Title/Abstract] OR "Triacylglycerols"[Title/Abstract])) OR "Triacylglycerol"[Title/Abstract]) AND ("cholesterol"[MeSH Terms] OR "HDL-Cholesterol"[Title/Abstract] OR "LDL-Cholesterol"[Title/Abstract] OR "HDL-Cholesterol"[Title/Abstract] OR "LDL-Cholesterol"[Title/Abstract] OR "high density lipoprotein"[Title/Abstract] OR "low density lipoprotein"[Title/Abstract] OR "lipid profile"[Title/Abstract] OR "cholesterol levels"[Title/Abstract])) OR "Epicholesterol"[Title/Abstract]) AND ((("fasted"[All Fields] OR "fasting"[MeSH Terms] OR "fasting"[All Fields] OR "fastings"[All Fields] OR "fasts"[All Fields]) AND "blood glucose"[MeSH Terms]) OR "fasting glucose"[Title/Abstract] OR "blood glucose levels"[Title/Abstract])) OR "glucose metabolism"[Title/Abstract]) AND ((("fasted"[All Fields] OR "fasting"[MeSH Terms] OR "fasting"[All Fields] OR "fastings"[All Fields] OR "fasts"[All Fields]) AND "insulin"[MeSH Terms]) OR "insulin levels"[Title/Abstract] OR "fasting serum insulin"[Title/Abstract] OR "insulin resistance"[Title/Abstract])) OR "insulin sensitivity"[Title/Abstract]) AND ("blood pressure"[MeSH Terms] OR "blood pressure determination"[MeSH Terms] OR "arterial pressure"[MeSH Terms] OR "systolic blood pressure"[Title/Abstract] OR "diastolic blood pressure"[Title/Abstract] OR "hypertension"[Title/Abstract] OR "high blood pressure"[Title/Abstract] OR "stroke"[Title/Abstract] OR "sbp"[Title/Abstract] OR "sbp"[Title/Abstract] OR "systolic bp"[Title/Abstract] OR "DBP"[Title/Abstract])) OR "diastolic bp"[Title/Abstract]) AND ("quality of life"[MeSH Terms] OR "health related quality of life"[Title/Abstract] OR "QoL"[Title/Abstract] OR "well-being"[Title/Abstract] OR "life satisfaction"[Title/Abstract] OR "functional status"[Title/Abstract] OR "SF-36"[Title/Abstract] OR "eortc qlq c30"[Title/Abstract] OR "WHOQOL"[Title/Abstract] OR "Fact-B"[Title/Abstract] OR "Fact-G"[Title/Abstract] OR "physical functioning"[Title/Abstract] OR "emotional functioning"[Title/Abstract] OR "social functioning"[Title/Abstract] OR "role functioning"[Title/Abstract] OR "cognitive functioning"[Title/Abstract] OR "global health status"[Title/Abstract] OR "mental health"[Title/Abstract] OR "bodily pain"[Title/Abstract])) OR "psychological well bieng"[Title/Abstract] |

2. **Web of Science**

| 1 | (((((((TS=(breast cancer)) OR TS=(breast neoplasm)) OR TS=(breast neoplasms)) OR TS=(neoplasm, breast)) OR TS=(breast tumor)) OR TS=(breast tumors)) OR TS=(breast carcinoma)) OR TS=(cancer patient) |
| --- | --- |
| 2 | (((((((((((((((((TS=(physical activity)) OR TS=(physical exercise)) OR TS=(exercise)) OR TS=(combined exercise)) OR TS=(combined training)) OR TS=(combined aerobic-resistance exercise)) OR TS=(aerobic exercise)) OR TS=(resistance exercise)) OR TS=(resistance training)) OR TS=(supervised training)) OR TS=(supervised exercise)) OR TS=(interventional training)) OR TS=(interventional exercise)) OR TS=(strength training)) OR TS=(exercise training)) OR TS=(aerobic AND resistance exercise)) OR TS=(aerobic AND strength training)) OR TS=(aerobic AND strength exercise) |
| 3 | (((TS=(metabolic syndrome)) OR TS=(metabolic biomarker)) OR TS=(metabolic marker)) OR TS=(metabolic markers) |
| 4 | (((((TS=(triglycerides)) OR TS=(triglyceride)) OR TS=(serum triglycerides)) OR TS=(Triacylglycerols)) OR TS=(Triacylglycerol)) |
| 5 | (((((TS=(cholesterol)) OR TS=(HDL-Cholesterol)) OR TS=(LDL-Cholesterol)) OR TS=(lipid profile)) OR TS=(cholesterol levels)) OR TS=(Epicholesterol) |
| 6 | (((TS=(fasting blood glucose)) OR TS=(fasting glucose)) OR TS=(blood glucose levels)) OR TS=(glucose metabolism) |
| 7 | ((((TS=(fasting insulin)) OR ALL=(insulin levels)) OR TS=(fasting serum insulin)) OR TS=(insulin resistance)) OR TS=(insulin sensitivity) |
| 8 | (((((((((TS=(blood pressure)) OR TS=(systolic blood pressure)) OR TS=(diastolic blood pressure)) OR TS=(hypertension)) OR TS=(high blood pressure)) OR TS=(stroke)) OR TS=(SBP)) OR TS=(DBP)) OR TS=(Systolic Bp)) OR TS=(Diastolic Bp) |
| 9 | ((((((((((((((((((TS=(quality of life)) OR TS=(health-related quality of life)) OR TS=(QoL)) OR TS=(well-being)) OR TS=(life satisfaction)) OR TS=(functional status)) OR TS=(SF-36)) OR TS=(EORTC QLQ-C30)) OR TS=(WHOQOL)) OR TS=(Fact-B)) OR TS=(Fact-G)) OR TS=(physical functioning)) OR TS=(emotional functioning)) OR TS=(social functioning)) OR TS=(role functioning)) OR TS=(cognitive functioning)) OR TS=(global health status)) OR TS=(mental health)) OR TS=(psychological well-being) |
| 10 | #3 OR #4 OR #5 OR #6 OR #7 OR #8 OR #9 |
|  | #1 AND #2 AND #10 |

3. **Medline**

| 1 | XB (("breast cancer" OR "breast neoplasm" OR "breast neoplasms" OR "neoplasm, breast" OR "breast tumor" OR "breast tumors" OR "breast carcinoma" OR "patient" OR "female" OR "survivor")) AND XB (("physical activity" OR "physical exercise" OR "exercise" OR "combined exercise" OR "combined training" OR "combined aerobic-resistance exercise" OR "aerobic exercise" OR "resistance exercise" OR "resistance training" OR "supervised training" OR "supervised exercise" OR "interventional training" OR "interventional exercise" OR "strength training" OR "exercise training" OR "aerobic AND resistance exercise" OR "aerobic AND strength training" OR "aerobic AND strength exercise")) AND XB (("metabolic syndrome" OR "metabolic biomarker" OR "metabolic marker" OR "metabolic markers" OR "metabolic syndrome" OR "triglycerides" OR "triglyceride" OR "serum triglycerides" OR "Triacylglycerols" OR "Triacylglycerol" OR "cholesterol" OR "HDL-Cholesterol" OR "LDL-Cholesterol" OR "lipid profile" OR "cholesterol levels" OR "Epicholesterol" OR "fasting blood glucose" OR "fasting glucose" OR "blood glucose levels" OR "glucose metabolism" OR "fasting insulin" OR "insulin levels" OR "fasting serum insulin" OR "insulin resistance" OR "insulin sensitivity" OR "blood pressure" OR "systolic blood pressure" OR "diastolic blood pressure" OR "hypertension" OR "high blood pressure" OR "stroke" OR "Sbp" OR "SBP" OR "Systolic Bp" OR "DBP" OR "Diastolic Bp" OR "quality of life" OR "health-related quality of life" OR "QoL" OR "well-being" OR "life satisfaction" OR "functional status" OR "SF-36" OR "EORTC QLQ-C30" OR "WHOQOL" OR "Fact-B" OR "Fact-G" OR "physical functioning" OR "emotional functioning" OR "social functioning" OR "role functioning" OR "cognitive functioning" OR "global health status" OR "mental health" OR "bodily pain" OR "psychological well-being")) |
| --- | --- |

4. **Cochrane Library**

| 1 | ("breast cancer"):ti,ab,kw OR (breast tumor):ti,ab,kw OR (breast tumors):ti,ab,kw OR (breast cancer patients):ti,ab,kw OR (Breast Malignant Neoplasm):ti,ab,kw in Cochrane Reviews |
| --- | --- |
| 2 | ("metabolic syndrome"):ti,ab,kw OR (metabolic biomarker):ti,ab,kw OR (metabolic marker):ti,ab,kw in Cochrane Reviews |
| 3 | ("triglyceride level"):ti,ab,kw OR (Triacylglycerols):ti,ab,kw OR ("triacylglycerol"):ti,ab,kw in Cochrane Reviews |
| 4 | ("cholesterol"):ti,ab,kw OR ("HDL cholesterol"):ti,ab,kw OR (LDL cholestrol):ti,ab,kw in Cochrane Reviews |
| 5 | ("fasting blood glucose level"):ti,ab,kw OR ("blood glucose level"):ti,ab,kw OR ("fasting glucose"):ti,ab,kw in Cochrane Reviews |
| 6 | ("fasting insulin level"):ti,ab,kw OR ("insulin level"):ti,ab,kw OR ("insulin-resistance"):ti,ab,kw OR ("insulin-resistances"):ti,ab,kw AND ("insulin sensitivity indices"):ti,ab,kw in Cochrane Reviews |
| 7 | ("blood pressure"):ti,ab,kw OR ("systolic blood pressure"):ti,ab,kw OR (diasystolic blood pressure):ti,ab,kw in Cochrane Reviews |
| 8 | ("Quality of Life Scale"):ti,ab,kw OR ("QOL"):ti,ab,kw OR ("Psychological General Well Being Index"):ti,ab,kw in Cochrane Reviews |
| 9 | ("physical activity"):ti,ab,kw OR ("physical exercise"):ti,ab,kw OR ("aerobic exercise"):ti,ab,kw OR ("resistance exercise"):ti,ab,kw in Cochrane Reviews |
| 10 | MeSH descriptor: [Exercise] explode all trees |
| 11 | MeSH descriptor: [Breast Neoplasms] explode all trees |
| 12 | MeSH descriptor: [Blood Pressure] explode all trees |
| 13 | MeSH descriptor: [Blood Glucose] explode all trees |
| 14 | MeSH descriptor: [Insulins] explode all trees |
| 15 | MeSH descriptor: [Triglycerides] explode all trees |
| 16 | MeSH descriptor: [Cholesterol] explode all trees |
| 17 | MeSH descriptor: [Lipoproteins] explode all trees |
| 18 | MeSH descriptor: [Quality of Life] explode all trees |
| 19 | #1 OR #11 |
| 20 | #10 OR #9 |
| 21 | #2 OR #3 OR #4 OR #5 OR #6 OR #7 OR #8 OR #12 OR #13 OR #14 OR #15 OR #16 OR #17 OR #18 |
|  | #19 AND #20 AND #21 |
