# Supplementary material for: The effects of combined aerobic and resistance exercise on metabolic blood biomarkers and quality of life in patients with breast cancer: a systematic review meta-analysis
Source: Front Oncol. 2026 Sep 11;16:1900945. doi: 10.3389/fonc.2026.1900945 (PMC13612194; doi:10.3389/fonc.2026.1900945)
Supplement: Supplementary file 2 [file DataSheet2.docx]

**The effects of combined aerobic and resistance exercise on metabolic blood biomarkers and quality of life in breast cancer patients: a systematic review and meta-analysis**

Contents

1.	Primary Analysis Results	...............................................................2

1.1.	Overall QoL	......................................................................................2

1.2.	Physical Functioning.........................................................................3

1.3.	Role Functioning	............................................................................4

1.4.	Emotional Functioning	..................................................................5

1.5.	Cognitive Functioning	..................................................................6

1.6.	Social Functioning	............................................................................7

2.	Metabolic Blood Biomarkers	.......................................................8

3.	Secondary Analysis	................................................................10

3.1.	Exercise Duration (duration/week)	...........................................10

3.1.1.	Overall QoL and Its Subscales	.....................................................10

3.1.2.	Metabolic Blood Biomarkers	.....................................................12

3.2.	Frequency (session/week)	.....................................................15

3.2.1.	Overall QoL and Its Subscales	.....................................................15

3.2.2.	Metabolic Blood Biomarkers	.....................................................17

3.3.	Session Duration (minutes/day)	.....................................................17

3.3.1.	Overall QoL and Its Subscales	.....................................................17

3.3.2.	Metabolic Blood Biomarkers	.....................................................20

4.	Subgroup analysis	..........................................................................22

**Supplementary material**

This supplementary file provides detailed results of meta-analysis, including forest and funnel plot, egger’s test, and trim-and-fill analysis for primary, secondary, and subgroup analysis.

# 1. **Primary Analysis Results**

## 1.1. Overall QoL

**Figure 1.** Funnel plot assessing publication bias for Overall QoL

**Figure 2.** Sensitivity analysis of Overall QoL

**Figure 3.** Egger's Regression of Overall QoL

**Figure 4.** Trim and Fill analysis of Overall QoL

## 1.2. Physical Functioning

**Figure 5.** Funnel plot assessing publication bias for Physical functioning

**Figure 6.** Egger's Regression of PF

**Figure 7.** Trim and Fill analysis of PF

## 1.3. Role Functioning

**Figure 8.** Funnel plot assessing publication bias for RF

**Figure 9.** Egger's Regression RF

## 1.4. Emotional Functioning

**Figure 10**. Forest plot showing the effect of combined exercise on emotional functioning in breast cancer

**Figure 11.** Funnel plot assessing publication bias for EF

**Figure 12.** Egger's Regression of EF

**Figure 13.** Trim and Fill analysis of EF

## 1.5. Cognitive Functioning

**Figure 14.** Funnel plot assessing publication bias for CF

**Figure 15.** Egger's Regression of CF

## 1.6. Social Functioning

**Figure 16** Forest plot showing the effect of combined exercise on social functioning in breast cancer

**Figure 17.** Funnel plot assessing publication bias for SF

**Figure 18.** Egger's Regression of SF

## 2. Metabolic Blood Biomarkers

**Figure 19.** Forest plot showing the effect of combined exercise on Glucose in breast cancer.

**Figure 20.** Forest plot showing the effect of combined exercise on Insulin in breast cancer.

**Figure 21.** Forest plot showing the effect of combined exercise on Triglycerides in breast cancer.

**Figure 22.** Forest plot showing the effect of combined exercise on LDL in breast cancer.

**Figure 23.** Forest plot showing the effect of combined exercise on SBP in breast cancer.

# 3. **Secondary Analysis**

## 3.1. Exercise Duration (duration/week)

### 3.1.1. Overall QoL and Its Subscales

**Figure 24.** Forest plot showing the effect of exercise duration per weeks on Overall QoL in breast cancer.

**Figure 25**. Forest plot showing the effect of exercise duration per weeks on FACT-B in breast cancer.

**Figure 26.** Forest plot showing the effect of exercise duration per weeks on physical functioning in breast cancer.

**Figure 27.** Forest plot showing the effect of exercise duration per weeks on role functioning in breast cancer.

**Figure 28.** Forest plot showing the effect of exercise duration per weeks on emotional functioning in breast cancer.

**Figure 29.** Forest plot showing the effect of exercise duration per weeks on cognitive functioning in breast cancer.

**Figure 30.** Forest plot showing the effect of exercise duration per weeks on social function in breast cancer.

### 3.1.2. Metabolic Blood Biomarkers

**Figure 31.** Forest plot showing the effect of exercise duration per weeks on Glucose in breast cancer.

**Figure 32.** Forest plot showing the effect of exercise duration per weeks on Insulin in breast cancer.

**Figure 33.** Forest plot showing the effect of exercise duration per weeks on HOMA-IR in breast cancer.

**Figure 34.** Forest plot showing the effect of exercise duration per weeks on Triglycerides in breast cancer.

**Figure 35.** Forest plot showing the effect of exercise duration per weeks on HDL in breast cancer.

**Figure 36.** Forest plot showing the effect of exercise duration per weeks on LDL in breast cancer.

**Figure 37.** Forest plot showing the effect of exercise duration per weeks on SBP in breast cancer.

**Figure 38.** Forest plot showing the effect of exercise duration per weeks on DBP in breast cancer.

## 3.2. Frequency (session/week)

### 3.2.1. Overall QoL and Its Subscales

**Figure 39.** Forest plot showing the effect of exercise Frequency on Overall QoL in breast cancer.

**Figure 40.** Forest plot showing the effect of exercise Frequency on PF in breast cancer.

**Figure 41.** Forest plot showing the effect of exercise Frequency on RF in breast cancer.

**Figure 42.** Forest plot showing the effect of exercise Frequency on EF in breast cancer.

**Figure 43.** Forest plot showing the effect of exercise Frequency on CF in breast cancer.

**Figure 44.** Forest plot showing the effect of exercise Frequency on SF in breast cancer.

### 3.2.2. Metabolic Blood Biomarkers

**Figure 45.** Forest plot showing the effect of exercise Frequency on Glucose in breast cancer.

**Figure 46.** Forest plot showing the effect of exercise Frequency on Insulin in breast cancer.

## 3.3. Session Duration (minutes/day)

### 3.3.1. Overall QoL and Its Subscales

**Figure 47.** Forest plot showing the effect of Session duration per day on Overall QoL in breast cancer.

**Figure 48.** Forest plot showing the effect of Session duration per day on FACT-B in breast cancer.

**Figure 49.** Forest plot showing the effect of Session duration per day on PF I breast cancer.

**Figure 50.** Forest plot showing the effect of Session duration per day on RF in breast cancer.

**Figure 51.** Forest plot showing the effect of Session duration per day on EF in breast cancer.

**Figure 52.** Forest plot showing the effect of Session duration per day on CF in breast cancer.

**Figure 53.** Forest plot showing the effect of Session duration per day on SF in breast cancer.

### 3.3.2. Metabolic Blood Biomarkers

**Figure 54.** Forest plot showing the effect of Session duration per day on Insulin in breast cancer.

**Figure 55.** Forest plot showing the effect of Session duration per day on HOMA-IR in breast cancer.

**Figure 56.** Forest plot showing the effect of Session duration per day on Triglycerides in breast cancer.

**Figure 57.** Forest plot showing the effect of Session duration per day on HDL in breast cancer.

**Figure 58.** Forest plot showing the effect of Session duration per day on SBP in breast cancer.

**Figure 59.** Forest plot showing the effect of Session duration per day on DBP in breast cancer.

# 4. **Subgroup analysis**

**Figure 60.** Forest Plot for subgroup by scales on Overall QoL

**Figure 61.** Forest Plot for subgroup by scales on PF

**Figure 62.** Forest Plot for subgroup by scales on RF

**Figure 63.** Forest Plot for subgroup by scales on EF

**Figure 64.** Forest Plot for subgroup by scales on CF

**Figure 65.** Forest Plot for subgroup by scales on SF

**Note:** All analyses were conducted using a random-effects model. Forest plots present pooled effect sizes (Hedges’ g) with 95% confidence intervals.
